# Bioinformatics Prediction of Polyketide Synthase Gene Clusters from *Mycosphaerella fijiensis*

**DOI:** 10.1371/journal.pone.0158471

**Published:** 2016-07-07

**Authors:** Roslyn D. Noar, Margaret E. Daub

**Affiliations:** 1 Department of Plant Pathology, North Carolina State University, Raleigh, North Carolina, 27695-7616, United States of America; 2 Department of Plant and Microbial Biology, North Carolina State University, Raleigh, North Carolina, 27695-7612, United States of America; Ruhr-University Bochum, GERMANY

## Abstract

*Mycosphaerella fijiensis*, causal agent of black Sigatoka disease of banana, is a Dothideomycete fungus closely related to fungi that produce polyketides important for plant pathogenicity. We utilized the *M*. *fijiensis* genome sequence to predict PKS genes and their gene clusters and make bioinformatics predictions about the types of compounds produced by these clusters. Eight PKS gene clusters were identified in the *M*. *fijiensis* genome, placing *M*. *fijiensis* into the 23rd percentile for the number of PKS genes compared to other Dothideomycetes. Analysis of the PKS domains identified three of the PKS enzymes as non-reducing and two as highly reducing. Gene clusters contained types of genes frequently found in PKS clusters including genes encoding transporters, oxidoreductases, methyltransferases, and non-ribosomal peptide synthases. Phylogenetic analysis identified a putative PKS cluster encoding melanin biosynthesis. None of the other clusters were closely aligned with genes encoding known polyketides, however three of the PKS genes fell into clades with clusters encoding alternapyrone, fumonisin, and solanapyrone produced by *Alternaria* and *Fusarium* species. A search for homologs among available genomic sequences from 103 Dothideomycetes identified close homologs (>80% similarity) for six of the PKS sequences. One of the PKS sequences was not similar (< 60% similarity) to sequences in any of the 103 genomes, suggesting that it encodes a unique compound. Comparison of the *M*. *fijiensis* PKS sequences with those of two other banana pathogens, *M*. *musicola* and *M*. *eumusae*, showed that these two species have close homologs to five of the *M*. *fijiensis* PKS sequences, but three others were not found in either species. RT-PCR and RNA-Seq analysis showed that the melanin PKS cluster was down-regulated in infected banana as compared to growth in culture. Three other clusters, however were strongly upregulated during disease development in banana, suggesting that they may encode polyketides important in pathogenicity.

## Introduction

*Mycosphaerella fijiensis* is the causal agent of black Sigatoka, also known as black leaf streak disease of banana and plantain. Black Sigatoka was first described in Fiji in 1963, and since then it has spread to most banana-growing regions around the world, including Latin America, Asia, Africa, and throughout the Pacific [1]. It has become one of the most important diseases of banana world-wide, causing up to 50% yield loss [2]. Infection of banana plants by *M*. *fijiensis* leads to necrotic streaking on leaves and loss of photosynthetic capacity [3]. The disease also causes premature fruit ripening, which is problematic for banana export companies, since bananas may over-ripen in transit [2]. Control of black Sigatoka is primarily through the extensive application of fungicides, which are estimated to account for 25–30% of the cost of production [1,2,3,4]. Development of fungicide-resistant strains is an on-going problem [3], and in developing countries, fungicide costs are prohibitive, and the disease results in severe yield losses [2].

Initial symptoms of the disease are chlorotic specks, followed by red-brown streaking, followed by the development of necrotic lesions surrounded by chlorotic halos and extensive blighting of the leaf tissue (S1 Fig) [1,2,3,5]. These symptoms (chlorosis, necrosis, streaking) as well as histological observations of symptoms ahead of hyphal colonization argue for the importance of toxins in disease development [5]. *M*. *fijiensis* is closely related to several fungi in the family Mycosphaerellaceae that are known to produce polyketide toxins important in pathogenicity. For example, *Cercospora* spp. produce the light-activated perylenequinone polyketide toxin, cercosporin, which kills host plant tissue through the production of reactive oxygen species; mutants deficient for the CTB1 polyketide synthase in the cercosporin biosynthetic pathway are reduced in both number and size of lesions on host plants [6]. *Ramularia collo-cygni* produces the anthraquinone toxin rubellin D. Although the role rubellin plays in the virulence of *R*. *collo-cygni* is not fully understood, it has been shown to generate reactive oxygen species in the light, resulting in fatty acid peroxidation and toxicity [7,8]. Also, *Dothistroma septosporum* has been shown to produce the polyketide dothistromin, which is structurally similar to a precursor of aflatoxin [9]. Dothistromin is important both for pathogenicity and for sporulation of the fungus [10].

The important role of polyketide toxins in related fungal pathogens has led to the investigation of possible production of polyketide toxins by *M*. *fijiensis*. A number of phytotoxic compounds have been identified from *M*. *fijiensis* culture filtrates, including several polyketides. These include fijiensin, 2,4,8-trihydroxytetralone, juglone, 4-hydroxyscytalone, and isoochracinic acid [11,12,13,14]. The compounds 2,4,8-trihydroxytetralone, 4-hydroxyscytalone and juglone are melanin shunt pathway metabolites [15,16,17,18]. These metabolites are toxic to banana tissue, and 2,4,8-trihydroxytetralone was shown to have host selectivity when comparing resistant versus susceptible banana varieties [11]. These results have implicated the melanin polyketide pathway as an important pathway for production of pathogenicity factors involved in disease development. In addition to toxic shunt metabolites, the melanin polyketide itself is known in many fungi to play important roles in pathogenicity, including appressorial function and entry into host tissue [19,20], hyphal tip protrusion [21,22], protection against plant defense compounds [23,24], and in some cases, production of reactive oxygen species [25] which may contribute to killing of host tissue later in the infection.

Recent sequencing of the *M*. *fijiensis* genome [26,27] allows for a bioinformatics analysis of PKS genes and clusters in the genome. Polyketide synthases in fungi are large, multi-domain enzymes that act iteratively to produce the polyketide chain. An iterative PKS enzyme must contain a ketosynthase domain (KS), an acyltransferase (AT) domain, and an acyl carrier protein domain (ACP) [28]. The reduction of the resulting polyketide is determined by three optional domains: ketoreductase (KR), dehydratase (DH), and enoyl reductase (ER) [28]. These three domains act sequentially to reduce a keto to a hydroxyl group, dehydrate a hydroxyl to an enoyl group, and reduce an enoyl to an alkyl group, respectively [29,30,31]. PKS enzymes lacking some or all of these domains will produce partially reduced or non-reduced polyketides.

The initial polyketide product may then be modified by other enzymes, typically encoded by genes that cluster together in the genome with the PKS gene [32,33]. These genes include methyltransferases, oxidoreductases, cytochrome P450s, transporters, and transcription factors [32]. While experimental data is necessary to confirm the involvement of a gene in the biosynthesis of a polyketide, predictions of genes in the cluster can be made based on proximity to the adjacent PKS and whether a gene is homologous to common types of secondary metabolite biosynthetic genes. Polyketide biosynthetic clusters can be small, as in the case of bikaverin from *Fusarium fujikuroi*, with an 18 kb cluster [34], or much larger, as for the aflatoxin biosynthetic cluster from *Aspergillus parasiticus* which spans 82 kb [35].

The main objectives of the work reported here were to predict PKS genes and their gene clusters in the published *M*. *fijiensis* genome, make bioinformatics predictions about the types of compounds that may be produced by these biosynthetic clusters, and identify PKS clusters with a possible role in pathogenicity by assaying expression in infected leaf tissue compared to expression during saprophytic growth in medium.

## Results

### Prediction of Polyketide Synthase Gene Clusters

Polyketide synthase genes from the *M*. *fijiensis* genome were identified using the program SMURF (Secondary Metabolite Unique Regions Finder), which is designed to identify secondary metabolite gene clusters from fungi [32]. SMURF identified one PKS gene on *M*. *fijiensis* scaffold 2 (*PKS2-1*), one on scaffold 7 (*PKS7-1*), a hybrid PKS-NRPS gene and three PKS genes on scaffold 8 (*PKS8-1*, *PKS8-2*, *Hybrid8-3*, and *PKS8-4*), and two PKS genes on scaffold 10 (*PKS10-1*, *PKS10-2*). PKS protein ID numbers are indicated in S1 Table. In addition to these PKS and hybrid PKS-NRPS genes, SMURF identified one PKS-like gene on scaffold 3.

### Comparison of Number of PKS Genes for Dothideomycete Fungi

**Fig 1:**
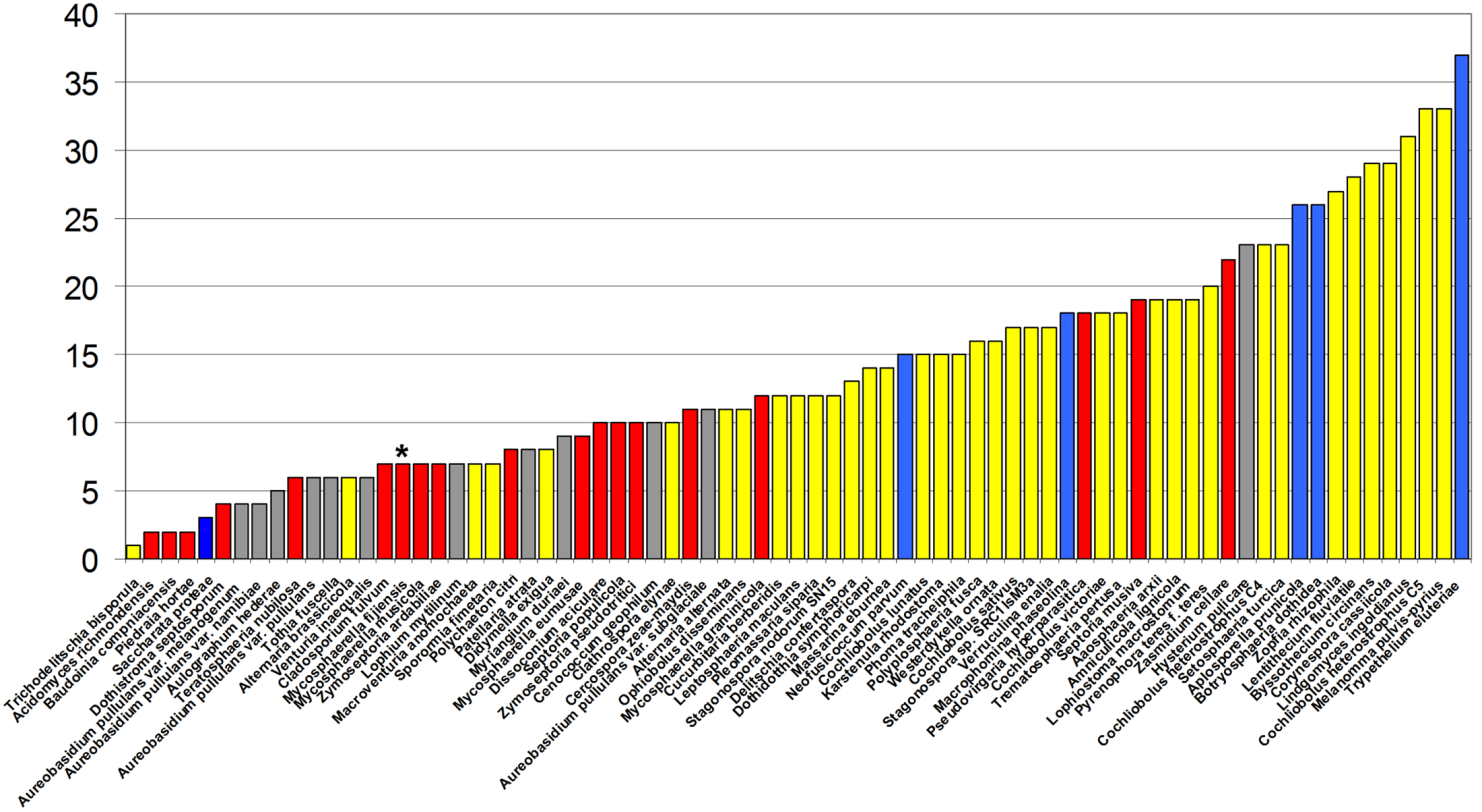
Number of PKS genes predicted from 75 Dothideomycete fungal genomes. The number of PKS genes predicted by the web tool SMURF (Secondary Metabolite Unique Regions Finder) from 75 Dothideomycete fungal genomes from JGI are shown. Red bars = Capnodiales; Yellow bars = Pleosporales; Blue bars = Botryosphaeriales; Gray bars = other orders within class Dothideomycetes. *M*. *fijiensis* is indicated with an asterisk above its bar.

Since production of polyketides is a common strategy for pathogenesis among necrotrophic and hemibiotrophic fungi, the repertoire of PKS genes in *M*. *fijiensis* was compared to that of other Dothideomycete fungi. SMURF was used to predict PKS genes from 74 additional Dothideomycete genomes available from Joint Genome Institute (JGI) and NCBI. This analysis revealed that the *M*. *fijiensis* genome falls into the 23rd percentile for number of PKS genes compared to the other Dothideomycete genomes and into the 34^th^ percentile for the number of PKS genes in the order Capnodiales, of which *M*. *fijiensis* is a member (Fig 1, S2 Table).

### Prediction of *M*. *fijiensis* PKS Domains

**Fig 2:**
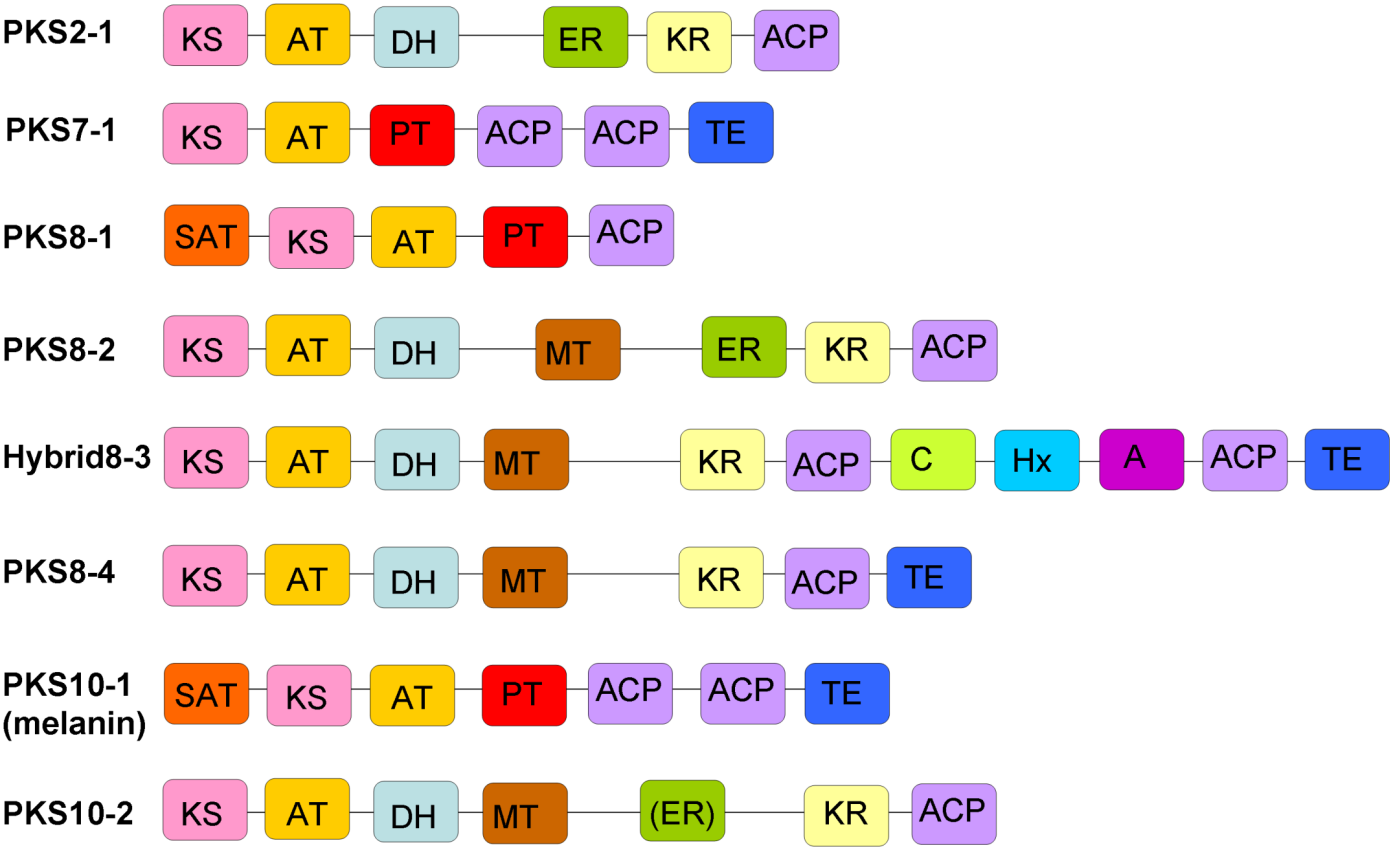
Domains predicted to be present in each *M*. *fijiensis* PKS or PKS-NRPS hybrid enzyme. PKS domains are: SAT = starter unit acyltransferase; KS = ketosynthase; AT = acyltransferase; DH = dehydratase; MT = methyltransferase; ER = enoyl reductase; KR = ketoreductase; PT = product template; ACP = acyl carrier protein domain; TE = thioesterase. NRPS domains are: C = condensation; Hx = HxxPF repeat domain; A = adenylation. Each type of domain is shown in a different color, to better display the similarities and differences between the domain organizations of different PKS enzymes. The ER domain of PKS10-2 is shown in parenthesis to indicate that it may or may not be functional, since the E-value for this domain is much lower than for ER domains of other PKS protein sequences (S3 Table).

Prediction of which domains are encoded in each PKS gene can provide clues as to whether the PKS enzyme has the necessary domains to be functional, and whether the PKS enzyme produces a reduced or a non-reduced product. Blastp analysis was done for all of the predicted PKS and PKS-like protein sequences, using the NCBI database. NCBI's Conserved Domain Database [36] predicted that the PKS-like protein encoded on scaffold 3 only contains a 3-oxoacyl-(acyl-carrier-protein) synthase domain, which confirms that it is unlikely to be a true PKS. Ketosynthase, acyltransferase, and phosphopantetheine attachment site (characteristic of acyl carrier protein and peptidyl carrier protein [37]) domains were predicted by the Conserved Domain Database to be included in each *M*. *fijiensis* PKS enzyme (Fig 2). Based on the optional domains predicted to be present in each *M*. *fijiensis* PKS enzyme, PKS7-1, PKS8-1, and PKS10-1 are likely to be non-reducing, since they do not contain KR, DH, or ER domains. PKS2-1 and PKS8-2 are likely to be highly reducing, since they contain all three reducing domains. Hybrid8-3, PKS8-4, and PKS10-2 are likely to be partially reducing, since they each contain KR and DH domains, but not complete ER domains. PKS10-2 does have a region with homology to an ER domain, but with a poor E-value when compared to E-values for ER domains in the other PKS enzymes (S3 Table).

### Prediction of Polyketide Biosynthetic Cluster Genes

**Fig 3:**
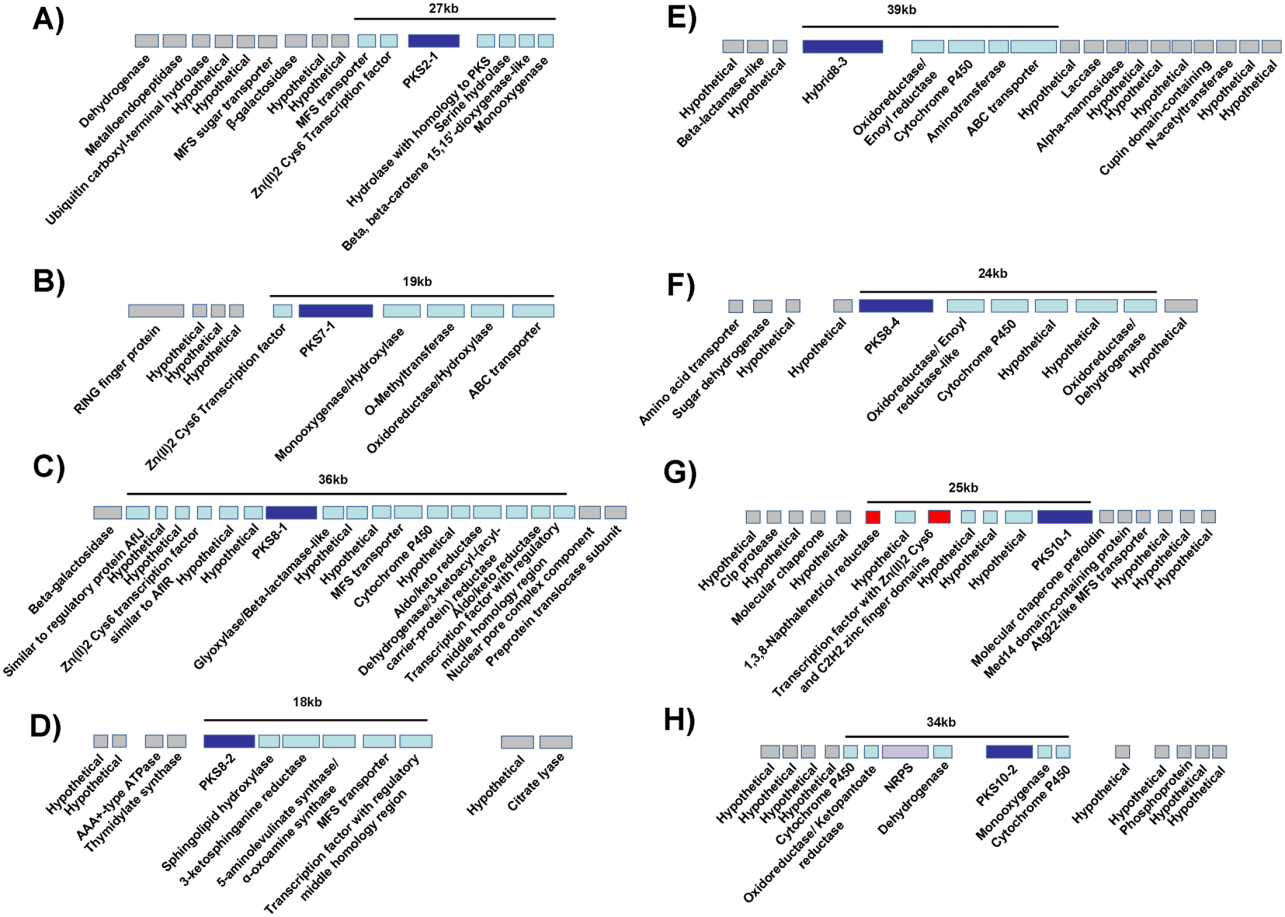
Proposed *M*. *fijiensis* PKS and PKS-NRPS gene clusters. The PKS or PKS-NRPS hybrid are shown, surrounded by the neighboring genes in the genome. Genes are labeled with putative functions of the corresponding proteins as determined by BLAST (S4 Table). PKS or PKS-NRPS genes are shown in dark blue, and NRPS genes are shown in purple, with other proposed biosynthetic cluster genes shown in light blue. Genes proposed to not be part of the biosynthetic cluster are shown in gray. For the melanin biosynthetic cluster, genes known to be part of melanin biosynthesis are shown in red. A) *PKS2-1* cluster; B) *PKS7-1* cluster; C) *PKS8-1* cluster; D) *PKS8-2* cluster; E) *Hybrid8-3* cluster; F) *PKS8-4* cluster; G) *PKS10-1* (melanin) cluster; H) *PKS10-2* cluster. No genes are shown to one side of the putative biosynthetic clusters for *PKS2-1* and *PKS7-1* because no gene models are found within 95 kb of the ones shown.

For each locus with a PKS gene in the *M*. *fijiensis* genome, BLAST searches were done for genes adjacent to the PKS to determine if they are homologous to genes common in secondary metabolite clusters. Starting at the PKS and working outward, genes were proposed to be part of the cluster if BLAST results indicated that the type of gene is commonly found in secondary metabolite clusters. Descriptions of the genes proposed for each of the eight PKS clusters are shown in S4 Table, including the GO, InterPro, and KOG descriptions of each gene, functional domains, homologs in other species, and links to NCBI and JGI descriptions. Once a region was found containing gene types not commonly found in clusters, these regions were proposed to flank the biosynthetic cluster. Genes and sizes of putative biosynthetic clusters and their flanking regions are shown graphically in Fig 3. A summary of the genes found in each proposed cluster is shown in Table 1.

**Table 1:** Predicted PKS and hybrid PKS-NRPS biosynthetic clusters.

	PKS 2–1 cluster	PKS 7–1 cluster	PKS 8–1 cluster	PKS 8–2 cluster	Hybrid 8–3 cluster	PKS 8–4 cluster	PKS 10–1 cluster	PKS 10–2 cluster
PKS	X	X	X	X		X	X	X
NRPS								X
Hybrid PKS-NRPS					X			
Methyltransferase		X						
Transporter	X	X	X	X	X			
Oxidoreductase	X	X	X	X	X	X	X	X
Cytochrome P450			X		X	X		X
Transcription factor	X	X	X	X			X	

For each PKS and hybrid PKS-NRPS gene predicted from *M*. *fijiensis* genome, presence or absence of nearby commonly found polyketide biosynthetic genes is indicated by an X.

### Identifying Homologs of *M*. *fijiensis* PKS Genes

**Fig 4:**
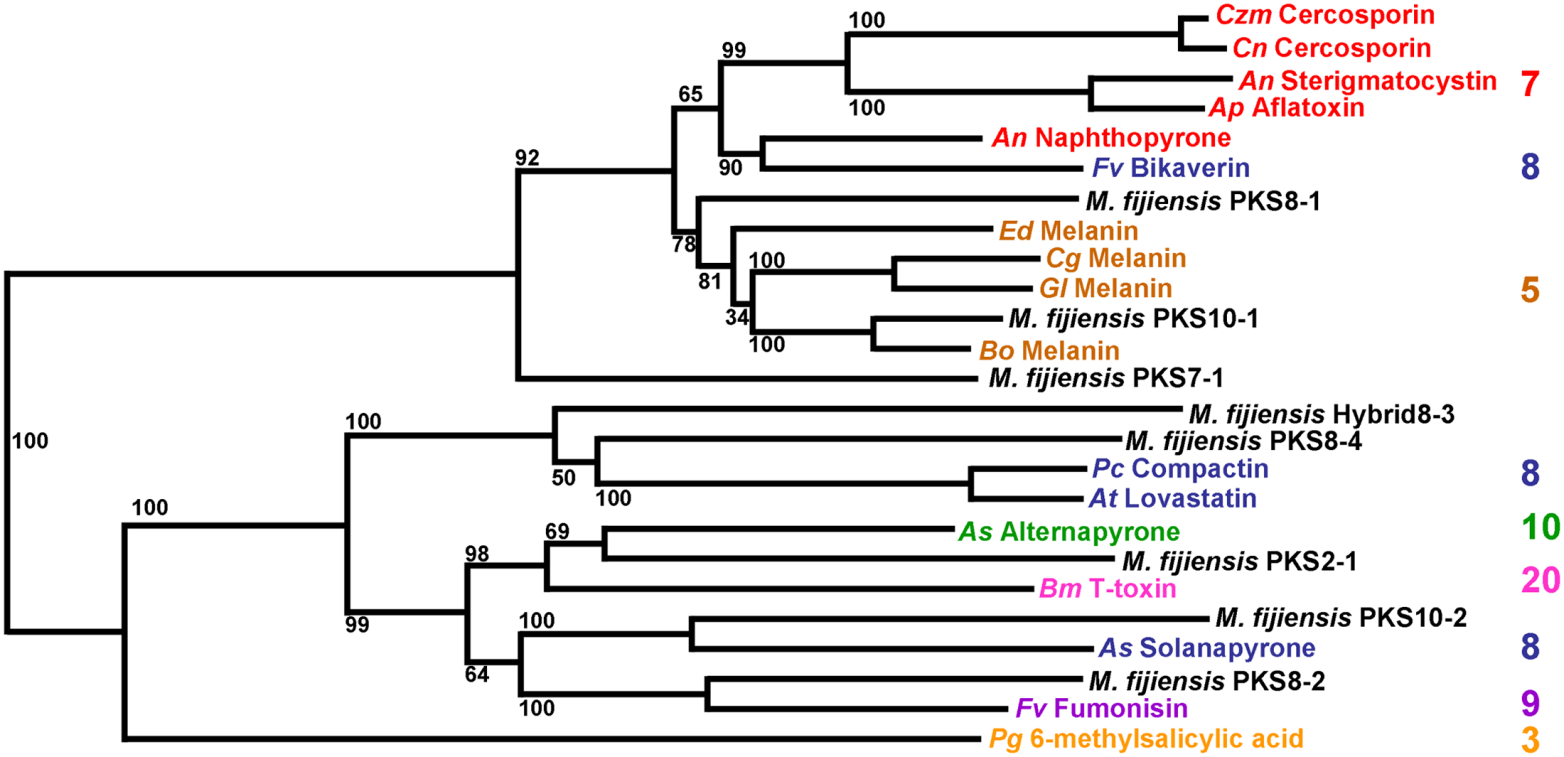
Maximum likelihood phylogenetic tree of PKS protein sequences. RAxML was used to generate a maximum likelihood phylogenetic tree of *M*. *fijiensis* PKS protein sequences as well as protein sequences from well-characterized PKS genes from other species. Products of each well-characterized PKS enzyme are indicated on the tree, along with abbreviations for species names. Numbers on branches indicate bootstrap support for the clade. Scale bar of branch length indicates substitutions per site. Number of iterations catalyzed by each PKS enzyme is indicated next to the name of the polyketide product of the PKS, in a matching color font. Orange = 3 ketide subunits; Brown = 5 ketide subunits; Red = 7 ketide subunits; Blue = 8 ketide subunits; Purple = 9 ketide subunits; Green = 10 ketide subunits; Pink = 20 ketide subunits. *An* = *Aspergillus nidulans*; *Ap* = *Aspergillus parasiticus*; *As* = *Alternaria solani*; *At* = *Aspergillus terreus*; *Bm* = *Bipolaris maydis*; *Bo* = *Bipolaris oryzae*; *Cg* = *Colletotrichum graminicola*; *Cn* = *Cercospora nicotianae*; *Czm* = *Cercospora zeae-maydis*; *Ed* = *Exophiala dermatitidis*; *Fv* = *Fusarium verticillioides; Gl* = *Glarea lozoyensis*; *Pc* = *Penicillium citrinum; Pg = Penicillium griseofulvum*. Accession numbers for each PKS sequence are available in S1 Table.

To determine if any of the PKS genes in *M*. *fijiensis* have close, well-characterized homologs, predicted protein sequences were aligned from *M*. *fijiensis* PKS genes and PKS genes from other fungi with well-characterized products. RAxML was used to generate a phylogenetic tree of the predicted PKS protein sequences (Fig 4). This analysis revealed that *M*. *fijiensis* PKS10-1 is in the same clade as PKS enzymes that produce DHN melanin from other fungi. None of the other PKS proteins from *M*. *fijiensis* were found to have close homology to characterized PKS proteins, but three fall into clades with excellent bootstrap values. PKS2-1 and the alternapyrone and T-toxin-producing PKS sequences form a clade with a bootstrap value of 98%. The alternapyrone PKS from *Alternaria solani* is the most similar to PKS2-1, with a sequence similarity of 58% when aligned using BLAST. PKS8-2 forms a clade with the fumonisin PKS from *Fusarium verticillioides*, with a bootstrap value of 100%, and has 58% similarity when aligned using BLAST. PKS10-2 forms a clade with the solanapyrone PKS from *Alternaria solani* with a bootstrap value of 100%, and has 52% similarity when aligned using BLAST. Percent similarity values between these three *M*. *fijiensis* PKS sequences and the characterized PKS sequences are lower than those between known orthologs (for example the cercosporin biosynthetic PKS is 91% similar between *C*. *nicotianae* and *C*. *zeae-maydis*, and the aflatoxin PKS from *Aspergillus parasiticus* and sterigmatocystin PKS from *A*. *nidulans* have 77% similarity), suggesting that they encode different products.

**Fig 5:**
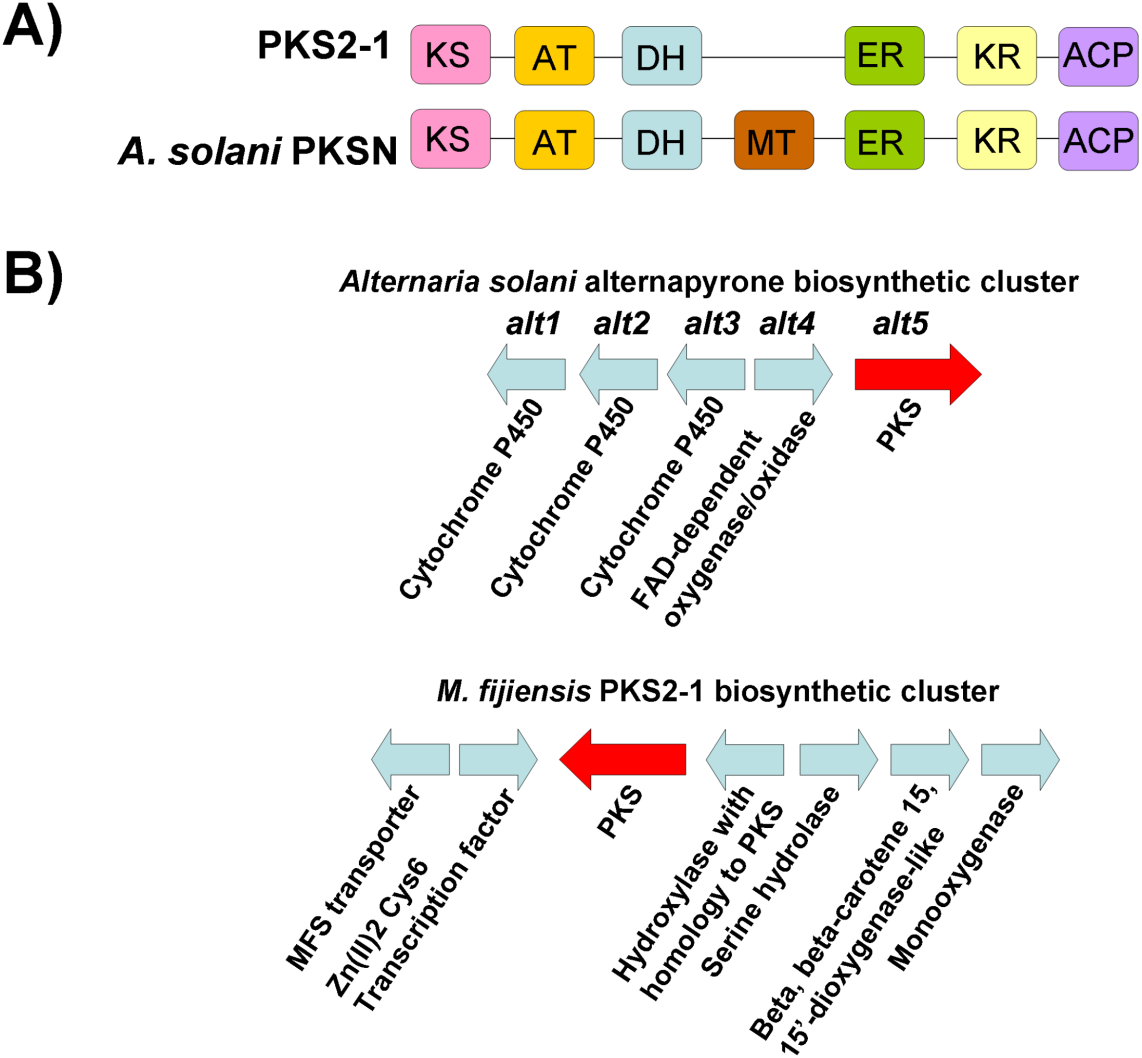
Comparison of domains and putative cluster genes for *M*. *fijiensis PKS2-1* and the alternapyrone-producing PKS. A) Domain organization of *M*. *fijiensis* PKS2-1 protein sequence, compared to domain organization of *A*. *solani* PKSN, which is encoded by the gene *alt5*. Each domain is shown in a different color. KS = ketosynthase (pink); AT = acyltransferase (orange); DH = dehydratase (blue); MT = methyltransferase (brown); ER = enoyl reductase (green); KR = ketoreductase (yellow); ACP = acyl carrier protein domain (purple). B) Putative biosynthetic cluster for *M*. *fijiensis PKS2-1* gene compared to alternapyrone biosynthetic cluster genes. Types of genes common between the two clusters being compared are shown in the same color. Types of genes that are different between the two clusters are shown in light blue.

In order to further characterize the similarities between the *M*. *fijiensis* PKS sequences and those of the characterized polyketides, we looked at domain structure as well as organization of the cluster. PKS2-1 formed a clade with a good bootstrap value to the alternapyrone and T-toxin-producing PKS sequences, with greatest similarity to the alternapyrone-producing PKSN protein from *Alternaria solani* (Fig 4). The *A*. *solani* PKSN has the following domain organization: KS-AT-DH-MT-ER-KR-ACP [38]. The *M*. *fijiensis* PKS2-1 has the same domain organization except that it lacks a MT domain (Fig 5A). Therefore, while alternapyrone is a highly methylated decaketide [38], the product of PKS2-1 may lack these methyl groups. Alternapyrone was identified as the product of PKSN by cloning the PKSN-encoding gene *alt5* from *A*. *solani* and expressing it in *Aspergillus oryzae* [38]. Since only the PKS was expressed in a heterologous system, the final product of the biosynthetic cluster in *A*. *solani* is currently unknown. There are genes for three cytochrome P450s and an oxidase adjacent to the *alt5* gene in the *A*. *solani* genome [38], which may modify alternapyrone to create a different metabolite. In the *M*. *fijiensis PKS2-1* biosynthetic cluster, *PKS2-1* is adjacent to several putative cluster genes such as a transporter and a transcription factor with a fungal Zn(2)-Cys(6) binuclear cluster domain, but not the cytochrome P450s or the oxidase that may be part of the *alt5* biosynthetic cluster (Fig 5B).

PKS8-2 forms a clade with the Fum1p fumonisin PKS protein sequence from *Fusarium verticillioides* (Fig 4). Fum1p has a protein sequence similarity of 72% with the AAL-toxin PKS Alt1p as well as an identical sequence of PKS domains: KS-AT-DH-MT-ER-KR-ACP [39]. The two PKS genes are also clustered with similar genes. The PKS enzymes produce similar, dimethylated, highly reduced polyketides, with the carbon backbone for AAL-toxin being 2 carbons shorter. Polyketide products for many PKS enzymes are released from the PKS via the thioesterase domain [40], however Fum1p and Alt1p do not contain thioesterase domains [39]. Instead, release of the 18-carbon chain for fumonisin and the 16-carbon chain for AAL-toxin is catalyzed via a α-oxoamine synthase, which condenses the polyketide chain with the α-carbon of L-alanine or glycine, respectively [39,41,42,43,44,45].

**Fig 6:**
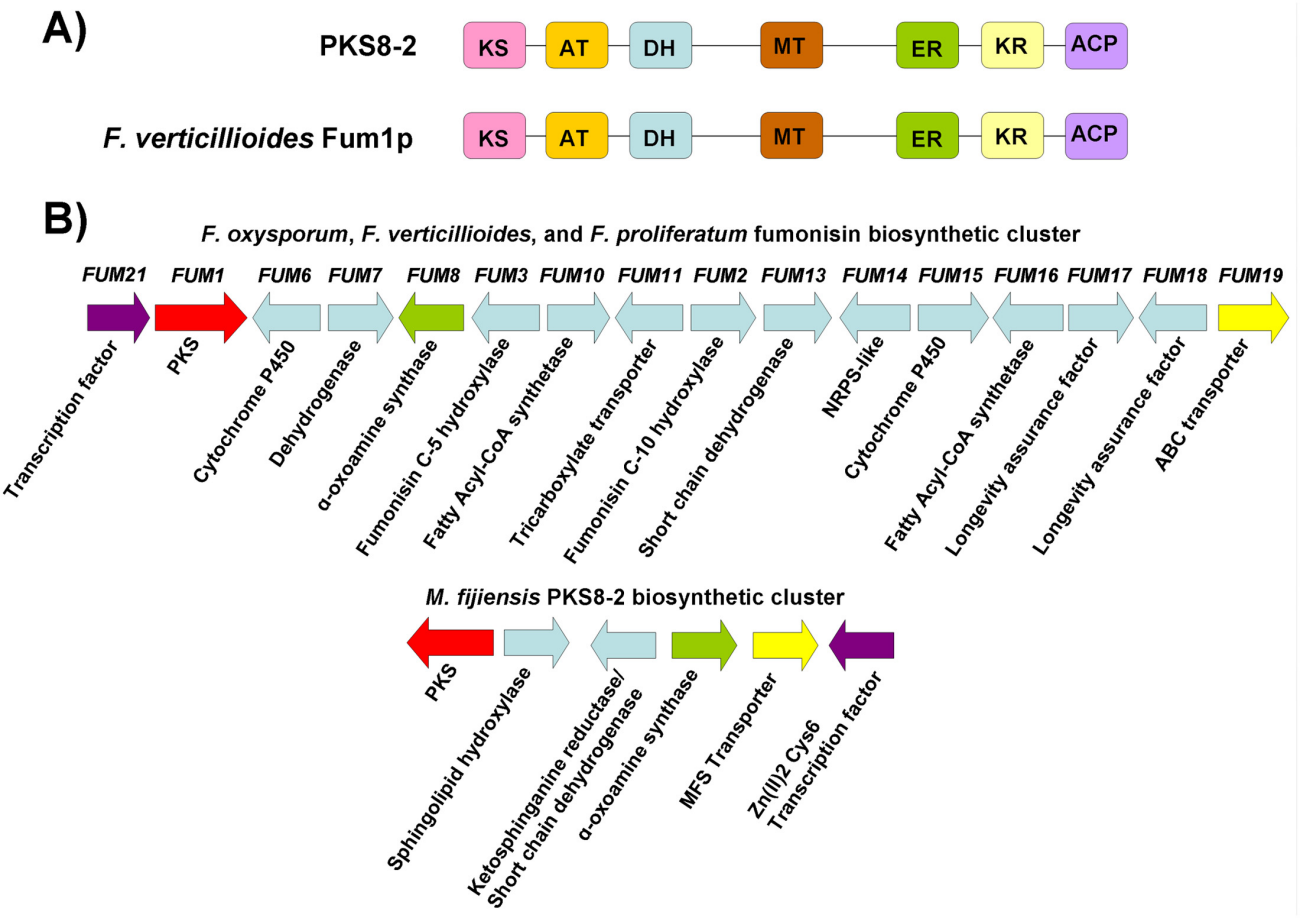
Comparison of domains and putative cluster genes for *M*. *fijiensis PKS8-2* and the fumonisin-producing PKS. A) Domain organization of *M*. *fijiensis* PKS8-2 protein sequence, compared to domain organization of *F*. *verticillioides* Fum1p. Each domain is shown in a different color. KS = ketosynthase (pink); AT = acyltransferase (orange); DH = dehydratase (blue); MT = methyltransferase (brown); ER = enoyl reductase (green); KR = ketoreductase (yellow); ACP = acyl carrier protein domain (purple). B) Putative biosynthetic cluster for *M*. *fijiensis* PKS8-2 gene compared to fumonisin biosynthetic cluster. Types of genes common between the two clusters being compared are shown in the same color. Types of genes that are different between the two clusters are shown in light blue.

Based on the domain analysis, PKS8-2 has the same domain organization as Fum1p and Alt1p (Fig 6A). Gene clusters were also compared for *PKS8-2* and *FUM1* (Fig 6B). Sixteen genes have been described from the fumonisin biosynthetic cluster, versus 6 genes predicted from the *M*. *fijiensis PKS8-2* cluster. Both clusters were predicted to contain a PKS, a transcription factor, a transporter, and an α-oxoamine synthase. For the genes with similar functions between these clusters, protein sequence similarity was determined by alignment with BLAST. While genes with similar predicted functions had significant alignment hits, sequence similarity was not especially high, with only the transcription factor having >60% sequence similarity (S5 Table).

**Fig 7:**
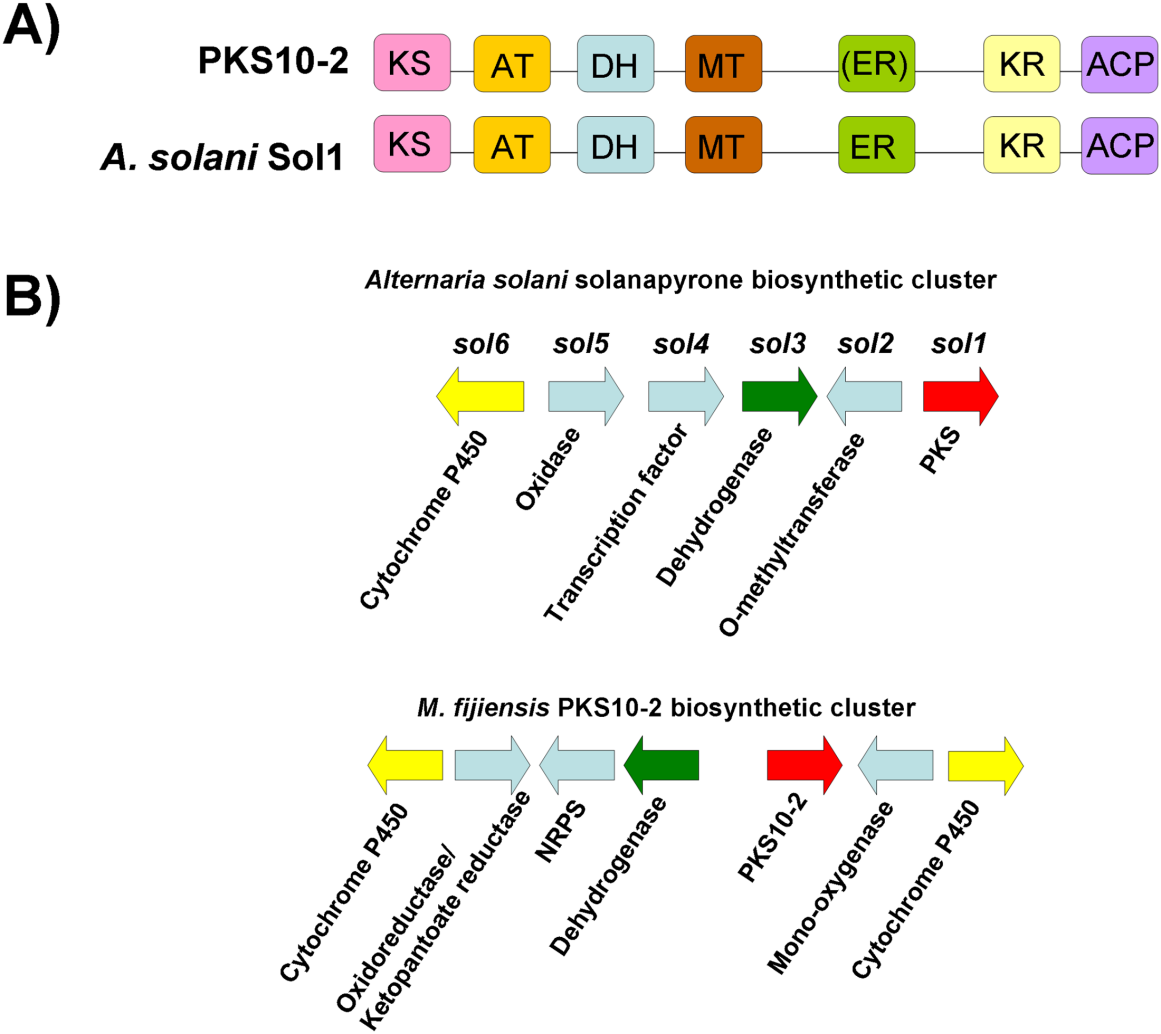
Comparison of domains and putative cluster genes for *M*. *fijiensis PKS10-2* and the solanapyrone-producing PKS. A) Domain organization of *M*. *fijiensis* PKS10-2 protein sequence, compared to domain organization of *A*. *solani* Sol1. Each domain is shown in a different color. KS = ketosynthase (pink); AT = acyltransferase (orange); DH = dehydratase (blue); MT = methyltransferase (brown); ER = enoyl reductase (green); KR = ketoreductase (yellow); ACP = acyl carrier protein domain (purple). The ER domain of PKS10-2 is shown with parentheses because while this domain was predicted using NCBI’s Conserved Domain Database, the E-value was very poor compared to E-values predicted for ER domains in other PKS sequences (S3 Table), and therefore it is uncertain whether the domain is functional. B) Putative biosynthetic cluster for *M*. *fijiensis PKS10-2* gene compared to the solanapyrone biosynthetic cluster. Types of genes common between the two clusters being compared are shown in the same color. Types of genes that are different between the two clusters are shown in light blue.

PKS10-2 formed a clade with good bootstrap support with the solanapyrone PKS Sol1 from *Alternaria solani*, which produces solanapyrone (Fig 4). Sol1 has a domain organization of: KS-AT-DH-MT-ER-KR-ACP [46]. PKS10-2 has a similar domain organization, except that while the E-value for the Sol1 ER domain is 3.69e-94, the E-value for the PKS10-2 ER domain is only 2.22e-05, and the Conserved Domain Database does not recognize the PKS10-2 ER active site (Fig 7A, S3 Table). Therefore it is not clear if the PKS10-2 ER domain is functional. Since the role of the ER domain in a polyketide synthase is to reduce a double bond to a single bond, the initial polyketide product of PKS10-2 may have an additional double bond that solanapyrone lacks. The solanapyrone cluster contains a PKS, an O-methyltransferase, a dehydrogenase, a transcription factor, a oxidase, and a cytochrome P450 [46]. The *PKS10-2* cluster is predicted to contain a PKS, a mono-oxygenase, two cytochrome P450s, an oxidoreductase with homology to ketopantoate reductase, a dehydrogenase, and a non-ribosomal peptide synthase (Fig 7B). While both clusters contain a cytochrome P450 and a dehydrogenase, they are otherwise very different, especially considering the PKS10-2 cluster is proposed to include a non-ribosomal peptide synthase (Fig 7B). Furthermore, while genes with similar predicted functions in the two clusters had significant alignment hits, sequence similarity was low, at 40% and 45% for the cytochrome P450 and the dehydrogenase, respectively (S5 Table).

### KS Modeling of Tertiary Structure

It has been shown that size of the substrate-binding cavity in the ketosynthase (KS) domain is important for determining the number of iterations [47]. Larger substrate binding cavities correlate with more iterations to form the polyketide product. KS domain cavities fall into three main size categories: small, medium, and large. For example, 6-methylsalicylic acid synthase (MSAS) and related PKS enzymes have a small substrate binding cavity of about 300 cubic angstroms, and they perform three iterations. Naphthopyrone (NAP) and related PKS enzymes have intermediate sized binding cavities of about 800 cubic angstroms, and perform five to eight iterations. The T-toxin PKS has the largest sized binding cavity, and it performs 20 iterations [47] (Fig 4).

Polyketide synthase and fatty acid synthase genes share similar domains and have a common evolutionary origin [48], and a crystal structure of the fatty acid synthesis enzyme 1kas is available [49]. Tertiary structure models comparing MSAS- and NAP-type PKS enzymes with the 1kas crystal structure reveals very similar structures. In each, two amino acid residues (positions 229 and 400 in 1kas) protrude into the substrate binding cavity, affecting its size. In MSAS-type PKS enzymes carrying out three iterations, these two amino acid residues are completely conserved (Y,Y). NAP-type PKS enzymes have Tyr and Ala residues in these positions, allowing their substrate binding cavity to be larger than that of MSAS [47].

Using the PKS/NRPS Analysis Website [50], KS domain sequences were predicted for each of the *M*. *fijiensis* PKS protein sequences, as well as for the other PKS sequences used for generation of the phylogenetic tree in Fig 4. These sequences were then aligned with the fatty acid synthesis protein sequence for 1kas. Using this alignment, amino acid residues corresponding to position 229 and 400 in 1kas were identified. Since PKS7-1, PKS8-1, and PKS10-1 were most closely related to NAP-type rather than MSAS-type or T-toxin-type PKS proteins (Fig 4), it was expected that they would have Tyr and Ala for the residues protruding into the active site. Indeed, Tyr and Ala were found to be in those two positions (S6 Table). The observation that PKS enzymes in the NAP-type clade have Tyr and Ala in these positions and all catalyze 5–8 iterations [47] is consistent with our observation that PKS7-1, PKS8-1, and PKS10-1 also have Tyr and Ala in these positions.

Since PKS2-1, PKS8-2, Hybrid8-3, PKS8-4, and PKS10-2 were more closely related to the T-toxin PKS than to the MSAS- or NAP-type PKS proteins (Fig 4), it is uncertain whether these two residues are informative. It is possible that the tertiary structure of their KS domains is different such that the two amino acid residues no longer line the cavity in the same way. However, PKS2-1 and PKS10-2 have the same two residues as the T-toxin and the solanapyrone PKS enzymes which catalyze 20 and 8 iterations respectively; PKS8-2 has the same two residues as the fumonisin PKS which catalyzes 9 iterations; and Hybrid8-3 and PKS8-4 have the same two residues as the compactin and lovastatin PKS enzymes, which both catalyze 8 iterations (S6 Table).

### Uncharacterized PKS Homologs in Dothideomycete Genomes

The phylogenetic analysis we conducted (Fig 4) only included sequences of characterized polyketide synthases with known products. In recent years, several genomes or partial genomes within the Mycosphaerellaceae have been sequenced and are publicly available including those of *Mycosphaerella musicola*, *Mycosphaerella eumusae*, *Mycosphaerella graminicola*, *Dothistroma septosporum*, *Septoria musiva*, *Pseudocercospora pini-densiflorae*, *Cercospora zeae-maydis*, *Mycosphaerella laricina*, and *Mycosphaerella arachidis*. Many of these are hemibiotrophic plant pathogens like *M*. *fijiensis* that are hypothesized to use toxic metabolites to facilitate pathogenesis. Since the range of polyketides produced by these fungi has not yet been fully characterized, it is possible that *M*. *fijiensis* produces similar metabolites as some of its close relatives.

Tblastn searches were done for each *M*. *fijiensis* PKS sequence against 103 Dothideomycete fungal genomes available from JGI and NCBI (S7 Table), and hits were arranged by bitscore to identify the closest homologs for each *M*. *fijiensis* PKS. Results are shown in Table 2. This analysis revealed that PKS2-1, PKS8-1, Hybrid8-3, PKS8-4, PKS10-1, and PKS10-2 all have close homologs with >80% sequence similarity in Mycosphaerellaceae genomes. By contrast, the best homologs of PKS7-1 have <60% sequence similarity, suggesting that this cluster may produce a unique product. Comparison of the *M*. *fijiensis* PKS sequences with those of the other two banana pathogens, *M*. *musicola* and *M*. *eumusae*, showed that these two species have close homologs to PKS2-1, Hybrid8-3, PKS10-1, and PKS10-2. By contrast, a PKS8-4 homolog was only found in *M*. *musicola*, and PKS7-1, PKS8-1, and PKS8-2 homologs were not found in either of the other species that infect banana.

**Table 2:** Top 10 tblastn hits by bitscore, for each *M*. *fijiensis* PKS.

**A)**					
***M*. *fijiensis* PKS**	**Species of BLAST hit**	**% identity**	**% similarity**	**bitscore**	**E-value**
PKS2-1	*Pseudocercospora pini-densiflorae*	92.51	95.29	4074	0
PKS2-1	*Mycosphaerella musicola*	92.53	95.72	4010	0
PKS2-1	*Mycosphaerella eumusae*	91.35	94.39	4008	0
PKS2-1	*Zymoseptoria passerinii*	70.26	80.76	2777	0
PKS2-1	*Mycosphaerella graminicola*	66.91	79.18	2140	0
PKS2-1	*Zymoseptoria pseudotritici*	67.6	79.46	2136	0
PKS2-1	*Zymoseptoria brevis*	66.91	79	2133	0
PKS2-1	*Zymoseptoria ardabiliae*	66.73	79.24	2120	0
PKS2-1	*Rhytidhysteron rufulum*	50.28	66.12	1475	0
PKS2-1	*Zasmidium cellare*	47.71	64.59	1043	0
**B)**					
***M*. *fijiensis* PKS**	**Species of BLAST hit**	**% identity**	**% similarity**	**bitscore**	**E-value**
PKS7-1	*Macroventuria anomochaeta*	36.02	55.48	1107	0
PKS7-1	*Lizonia empirigonia*	35.83	55.59	1107	0
PKS7-1	*Delitschia confertaspora*	36.05	54.69	1101	0
PKS7-1	*Didymella exigua*	36.34	55.2	1097	0
PKS7-1	*Hysterium pulicare*	36.25	54.67	1095	0
PKS7-1	*Pleomassaria siparia*	35.88	55.11	1095	0
PKS7-1	*Didymella zeae-maydis*	35.76	54.8	1094	0
PKS7-1	*Sporormia fimetaria*	37.51	55.03	1092	0
PKS7-1	*Elsinoe ampelina*	36.19	54.18	1091	0
PKS7-1	*Melanomma pulvis-pyrius*	35.98	54.82	1090	0
**C)**					
***M*. *fijiensis* PKS**	**Species of BLAST hit**	**% identity**	**% similarity**	**bitscore**	**E-value**
PKS8-1	*Pseudocercospora pini-densiflorae*	89.78	92.44	2572	0
PKS8-1	*Byssothecium circinans*	69.53	81.47	2416	0
PKS8-1	*Passalora fulva*	61.84	76	2283	0
PKS8-1	*Cladosporium fulvum*	61.84	76	2283	0
PKS8-1	*Pyrenophora tritici-repentis*	65.36	77.94	2222	0
PKS8-1	*Pyrenophora teres f*. *teres*	64.95	77.98	2217	0
PKS8-1	*Septoria populicola*	66.13	78.08	2083	0
PKS8-1	*Septoria musiva*	65.88	77.89	2083	0
PKS8-1	*Trematosphaeria pertusa*	60.92	75.53	2038	0
PKS8-1	*Cercospora canescens*	66.5	78.04	1942	0
**D)**					
***M*. *fijiensis* PKS**	**Species of BLAST hit**	**% identity**	**% similarity**	**bitscore**	**E-value**
PKS8-2	*Zymoseptoria pseudotritici*	45.14	63.39	2043	0
PKS8-2	*Mycosphaerella graminicola*	45.3	63.19	2035	0
PKS8-2	*Zymoseptoria passerinii*	45.21	63.3	2030	0
PKS8-2	*Aulographum hederae*	51.35	68.61	1892	0
PKS8-2	*Lindgomyces ingoldianus*	43.28	61.17	1828	0
PKS8-2	*Decorospora gaudefroyi*	45.09	62.41	1698	0
PKS8-2	*Zasmidium cellare*	40.6	58.46	1622	0
PKS8-2	*Polyplosphaeria fusca*	40.05	58.29	1620	0
PKS8-2	*Pleomassaria siparia*	42.61	62.21	1585	0
PKS8-2	*Amniculicola lignicola*	39.29	57	1584	0
**E)**					
***M*. *fijiensis* PKS**	**Species of BLAST hit**	**% identity**	**% similarity**	**bitscore**	**E-value**
Hybrid8-3	*Pseudocercospora pini-densiflorae*	83.96	89.59	6688	0
Hybrid8-3	*Mycosphaerella eumusae*	86.18	91.32	4383	0
Hybrid8-3	*Mycosphaerella musicola*	85.07	91.06	4315	0
Hybrid8-3	*Cercospora zeae-maydis*	69.61	81.3	3565	0
Hybrid8-3	*Cercospora canescens*	68.93	80.92	3553	0
Hybrid8-3	*Septoria populicola*	67.38	78.76	3441	0
Hybrid8-3	*Passalora fulva*	66.15	78.59	3388	0
Hybrid8-3	*Cladosporium fulvum*	66.15	78.59	3388	0
Hybrid8-3	*Mysosphaerella sp*. *Ston1*	67.5	79.59	3302	0
Hybrid8-3	*Mycosphaerella arachidis*	65.39	77.82	3267	0
**F)**					
***M*. *fijiensis* PKS**	**Species of BLAST hit**	**% identity**	**% similarity**	**bitscore**	**E-value**
PKS8-4	*Mycosphaerella musicola*	80.84	88.21	2907	0
PKS8-4	*Pyrenophora teres f*. *teres*	47.77	63.93	2460	0
PKS8-4	*Lizonia empirigonia*	42.25	60.09	2120	0
PKS8-4	*Alternaria alternata*	45.2	63.2	2028	0
PKS8-4	*Glonium stellatum*	46.56	64.8	1823	0
PKS8-4	*Pyrenophora tritici-repentis*	49.92	65.89	1746	0
PKS8-4	*Setosphaeria turcica*	49.77	66.6	1723	0
PKS8-4	*Pseudovirgaria hyperparasitica*	38.77	56.7	1558	0
PKS8-4	*Stagonospora sp*. SRC1lsM3a	38.86	57.64	1553	0
PKS8-4	*Cenococcum geophilum*	39.29	57.45	1523	0
**G)**					
***M*. *fijiensis* PKS**	**Species of BLAST hit**	**% identity**	**% similarity**	**bitscore**	**E-value**
PKS10-1	*Pseudocercospora pini-densiflorae*	97.65	98.64	4080	0
PKS10-1	*Mycosphaerella musicola*	95.48	97.65	4012	0
PKS10-1	*Mycosphaerella eumusae*	95.75	97.06	4011	0
PKS10-1	*Mycosphaerella laricina*	83.71	90.6	3604	0
PKS10-1	*Passalora fulva*	83.94	90.47	3575	0
PKS10-1	*Cladosporium fulvum*	83.94	90.47	3575	0
PKS10-1	*Mycosphaerella arachidis*	82.82	89.68	3565	0
PKS10-1	*Cercospora zeae-maydis*	83.21	89.85	3551	0
PKS10-1	*Dothistroma septosporum*	82.68	90.15	3549	0
PKS10-1	*Mysosphaerella sp*. *Ston1*	81.83	88.25	3534	0
**H)**					
***M*. *fijiensis* PKS**	**Species of BLAST hit**	**% identity**	**% similarity**	**bitscore**	**E-value**
PKS10-2	*Mycosphaerella eumusae*	80.87	89.18	1412	0
PKS10-2	*Mycosphaerella musicola*	80.48	88.01	1398	0
PKS10-2	*Mycosphaerella sp*. *Ston1*	43.58	59.15	1282	0
PKS10-2	*Melanomma pulvis-pyrius*	33.02	49.34	1163	0
PKS10-2	*Pyrenochaeta sp*. DS3sAY3a	32.95	50.28	1160	0
PKS10-2	*Cochliobolus carbonum*	35.72	51.89	1035	0
PKS10-2	*Stagonospora sp*. SRC1lsM3a	33.55	50.45	1034	0
PKS10-2	*Setosphaeria turcica*	32.09	48.07	984	0
PKS10-2	*Cucurbitaria berberidis*	35.08	52.77	984	0
PKS10-2	*Aureobasidium pullulans* var. *subglaciale*	42.68	58.86	937	0

Table indicates the *M*. *fijiensis* PKS, species where the blast hit was found, percent sequence identity, percent sequence similarity, bitscore, and E-value. Hits are arranged in the table by bitscore. A) PKS2-1; B) PKS7-1; C) PKS8-1; D) PKS8-2; E) Hybrid 8–3; F) PKS8-4; G) PKS10-1; H) PKS10-2.

**Fig 8:**
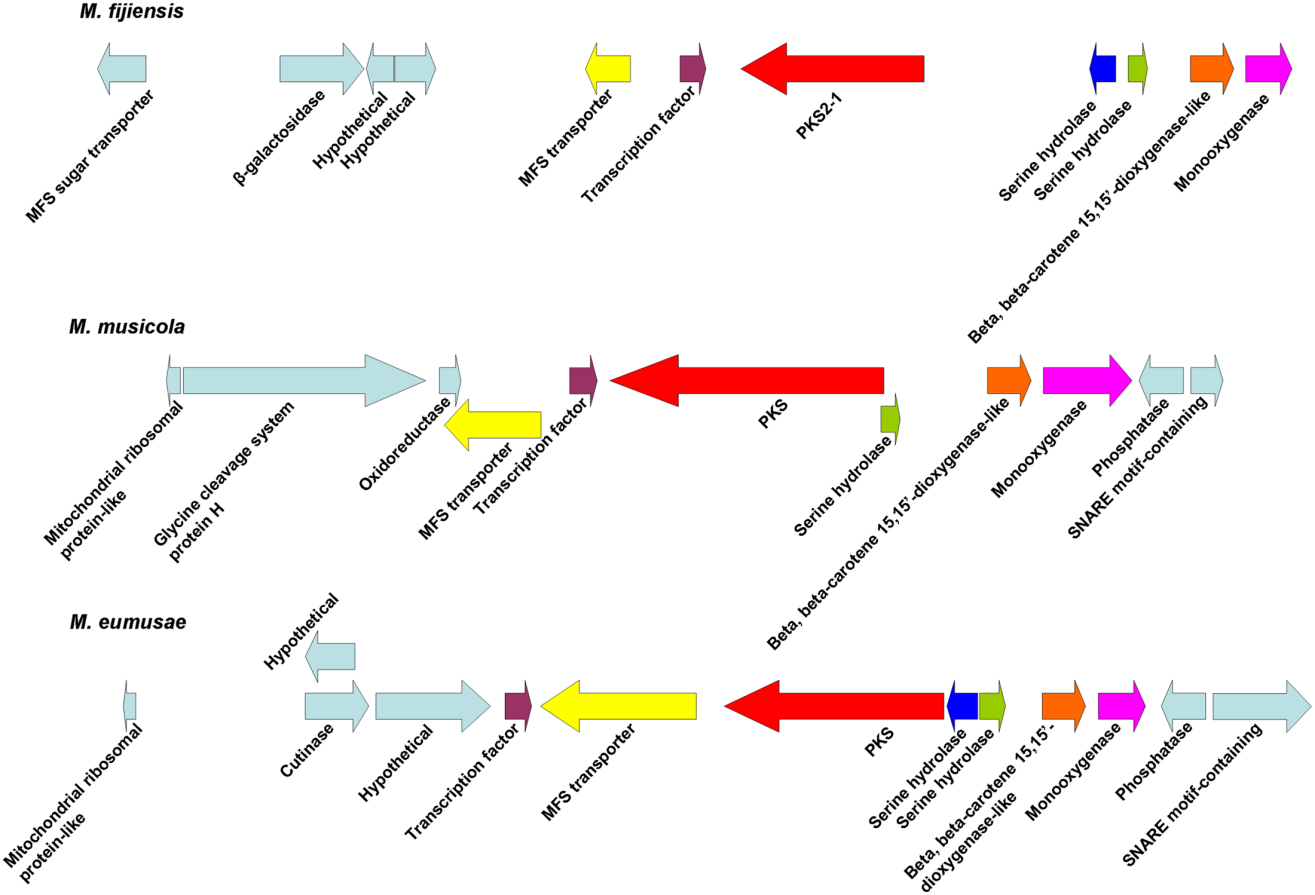
Comparison of *PKS2-1* gene clusters from *Mycosphaerella* species. Putative biosynthetic cluster for *M*. *fijiensis PKS2-1* gene compared to the orthologous cluster in *M*. *musicola* and *M*. *eumusae*. Putative orthologous genes are shown in the same color. Genes flanking the putative biosynthetic cluster are shown in light blue.

**Fig 9:**
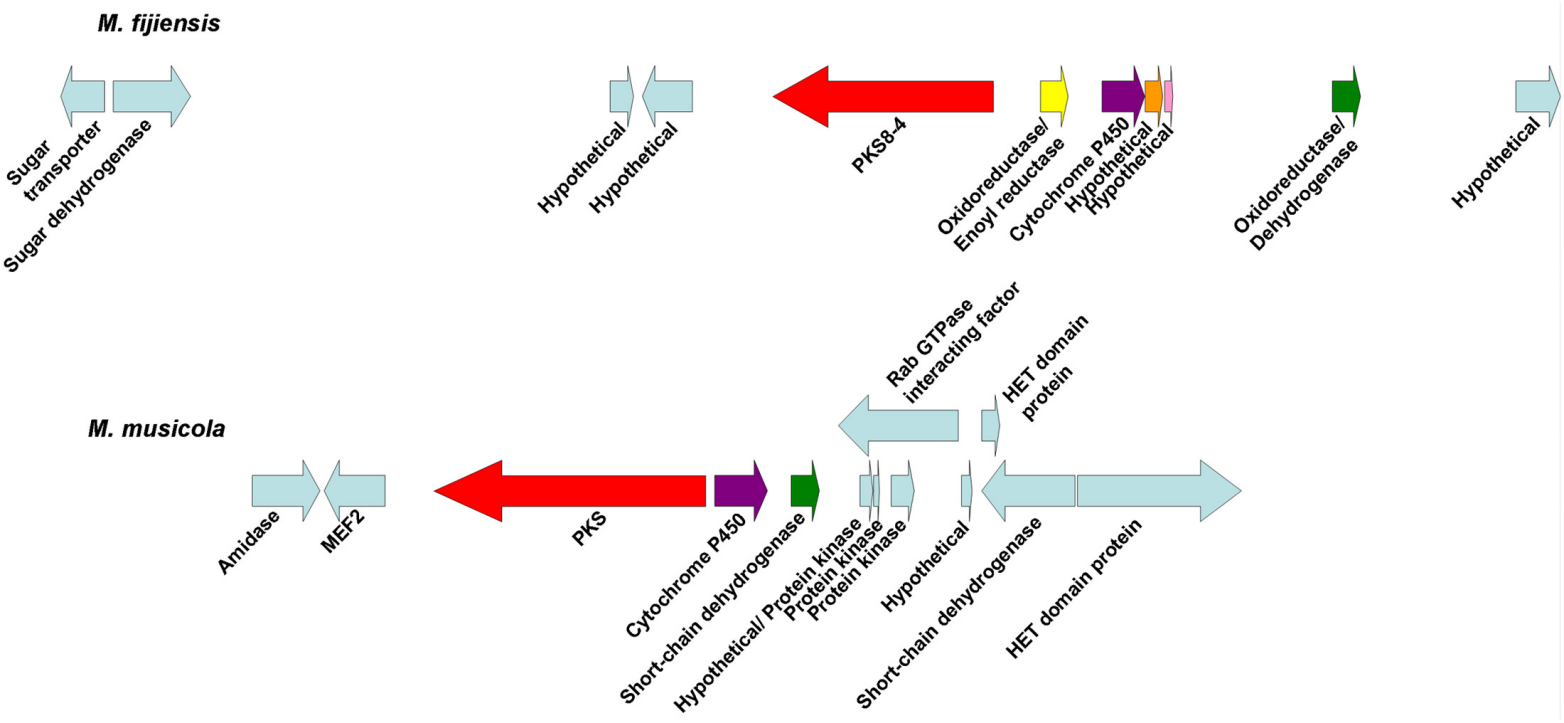
Comparison of *PKS8-4* gene clusters from *M*. *fijiensis* and *M*. *musicola*. Putative biosynthetic cluster for *M*. *fijiensis PKS8-4* gene compared to the orthologous cluster in *M*. *musicola*. Putative orthologous genes are shown in the same color. Genes flanking the putative biosynthetic cluster are shown in light blue.

**Fig 10:**
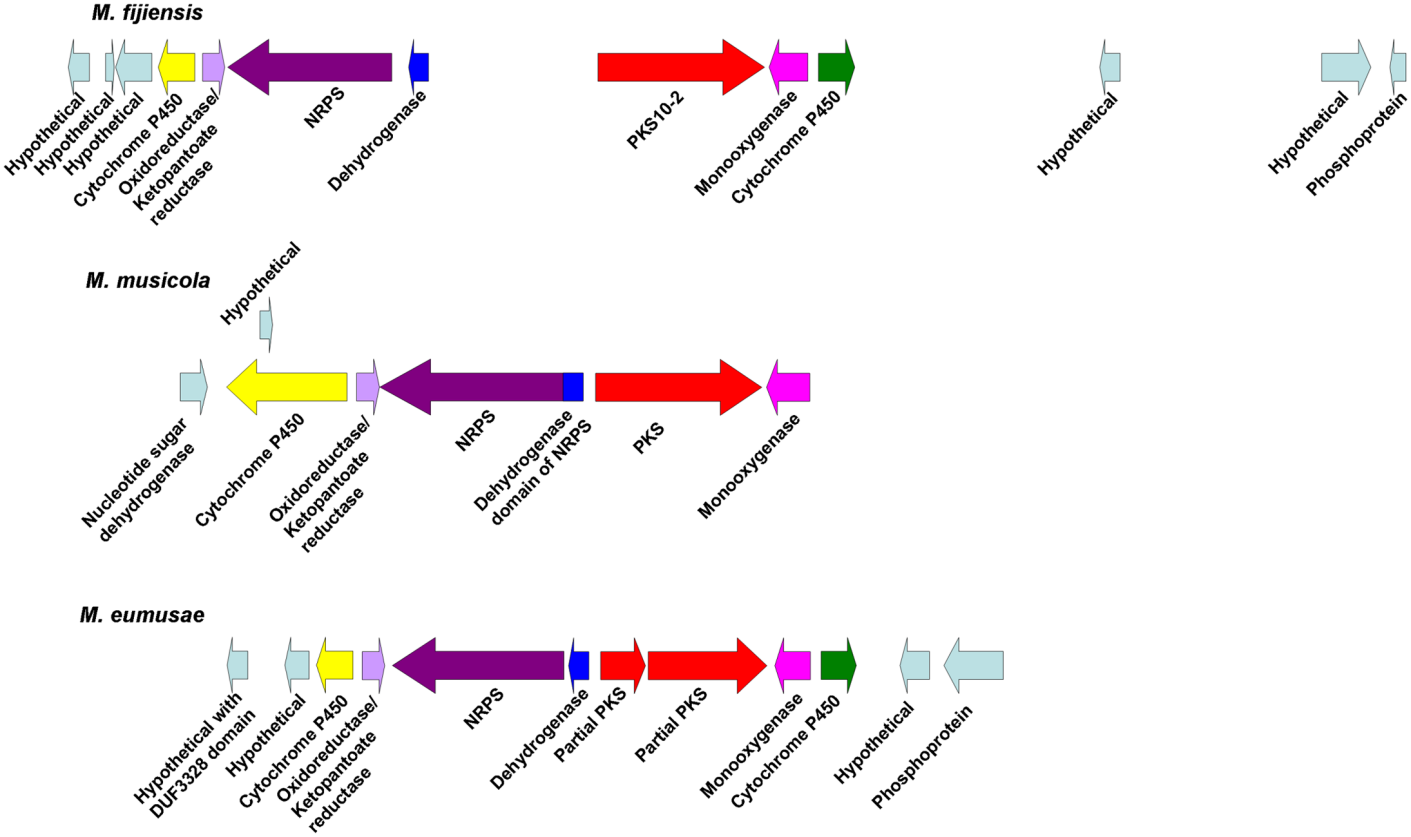
Comparison of *PKS10-2* gene clusters from *Mycosphaerella* species. Putative biosynthetic cluster for *M*. *fijiensis PKS10-2* gene compared to the orthologous cluster in *M*. *musicola* and *M*. *eumusae*. Putative orthologous genes are shown in the same color. Genes flanking the putative biosynthetic cluster are shown in light blue.

Since the polyketide synthases PKS2-1, PKS8-4, and PKS10-2 each had close homologs in the related banana pathogens *M*. *musicola* and *M*. *eumusae* (Table 2), we compared the biosynthetic clusters for each of the corresponding PKS genes in the different species. Conserved domains were predicted for proteins encoded by genes neighboring the PKS gene in each species (S8 Table). For sequences with similar predicted functions between species, a blastp search was done for each *M*. *fijiensis* protein sequence to determine percent identity and similarity with its ortholog in the other species (S9 Table). This analysis revealed that the biosynthetic clusters are extremely similar for *PKS2-1*. Clusters for all three species share genes encoding a PKS, an MFS transporter, a transcription factor, a monooxygenase, a dioxygenase, and at least one serine hydrolase (Fig 8), and most of the orthologous protein sequences have >90% similarity (S9 Table). For the *PKS8-4* gene cluster, both *M*. *fijiensis* and *M*. *musicola* have genes encoding a PKS, a cytochrome P450, and a dehydrogenase (Fig 9). However, the *M*. *fijiensis* gene cluster contains a gene encoding an enoyl reductase-like protein which *M*. *musicola* lacks, and the *M*. *musicola* gene cluster contains a second dehydrogenase gene that *M*. *fijiensis* lacks (Fig 9). The *PKS8-4* cluster proteins that the two species share all have at least 83% sequence similarity (S9 Table). All three species have nearly identical biosynthetic clusters for *PKS10-2* (Fig 10). All three species have genes encoding a PKS, an NRPS, a dehydrogenase, a monooxygenase, a cytochrome P450, and an oxidoreductase with similarity to ketopantoate reductase. In *M*. *fijiensis* and *M*. *eumusae*, the dehydrogenase is predicted to be encoded by a stand-alone gene, whereas in *M*. *musicola*, it is predicted to be a part of the NRPS (Fig 10, S9 Table). *M*. *fijiensis* and *M*. *eumusae* have a second cytochrome P450 gene distal to the monooxygenase. The *M*. *musicola* scaffold ends just after the monooxygenase gene, and a close homolog of this cytochrome P450 is found at the end of another scaffold. It may be that greater sequencing depth of the *M*. *musicola* genome would reveal that these two scaffolds are in fact part of the same chromosome. Regardless, all proteins encoded by the *M*. *fijiensis PKS10-2* biosynthetic cluster have orthologs with high sequence similarity (at least 70%) in these two other banana pathogens.

### Expression of *M*. *fijiensis* PKS Genes during Colonization of Banana

**Fig 11:**
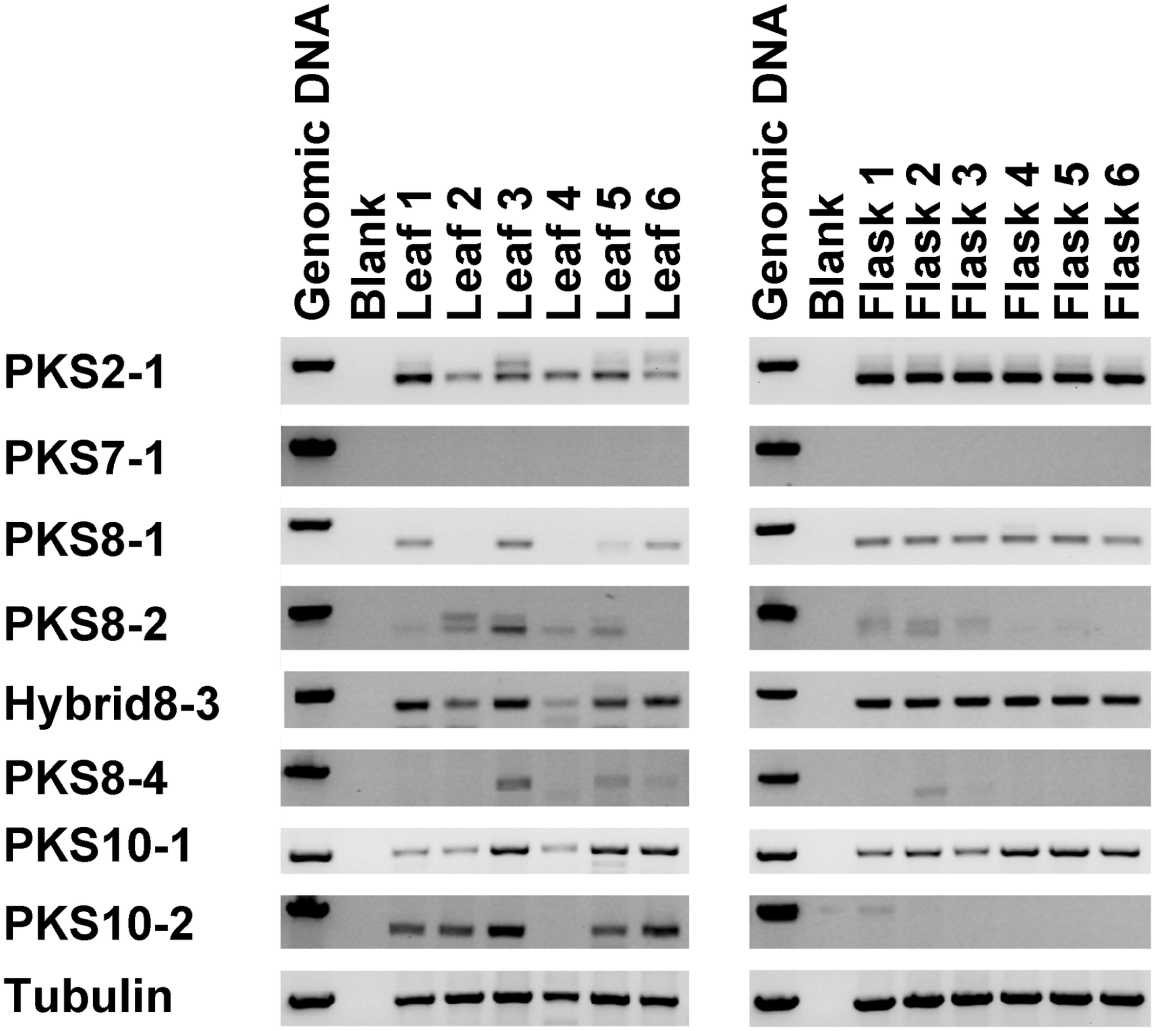
RT-PCR analysis of PKS gene expression in infected leaves and mycelial cultures. *M*. *fijiensis* isolate 10CR1-24 was used to infect banana tissue-culture plants, as well as to inoculate PDB medium. RT-PCR assays for each PKS or hybrid PKS-NRPS were performed on the resulting leaf lesions or mycelium from the PDB liquid culture. Where possible (for all genes except beta-tubulin and *PKS10-1*), primers were designed to span an intron, to distinguish cDNA from any gDNA contamination. Genomic DNA isolated from 10CR1-24 was used as a positive control, and reactions were also done with no added template, as a negative control. Each lane represents RNA isolated from individual infected leaves (left) or mycelium grown in flask culture (right).

We hypothesized that PKS genes important for pathogenicity should be expressed during infection of host banana tissue. To identify PKS genes that are expressed during infection, we first compared expression of the *M*. *fijiensis* PKS genes in inoculated leaves of in vitro-cultured banana plants as compared to mycelial cultures using RT-PCR analysis. Isolate 10CR1-24 was used to inoculate leaves of tissue cultured plants, and leaves were harvested at 5 weeks post-inoculation, once they had become symptomatic (S1C Fig). As a control, isolate 10CR1-24 was grown in liquid PDB medium, and mycelium was harvested after 1 week incubation. RT-PCR assays were performed using tissue from both conditions (Fig 11). This analysis revealed that *Hybrid8-3*, *PKS2-1*, and *PKS8-1* were most strongly expressed in culture, with less expression in infected leaf tissue. *PKS8-2* and *PKS8-4* were expressed more often in infected leaves than in culture, and *PKS10-2* was strongly expressed in the infected leaf samples, but not expressed in the flask samples. *PKS10-1* was expressed under both conditions, and *PKS7-1* expression was not detected in any samples in either condition.

**Fig 12:**
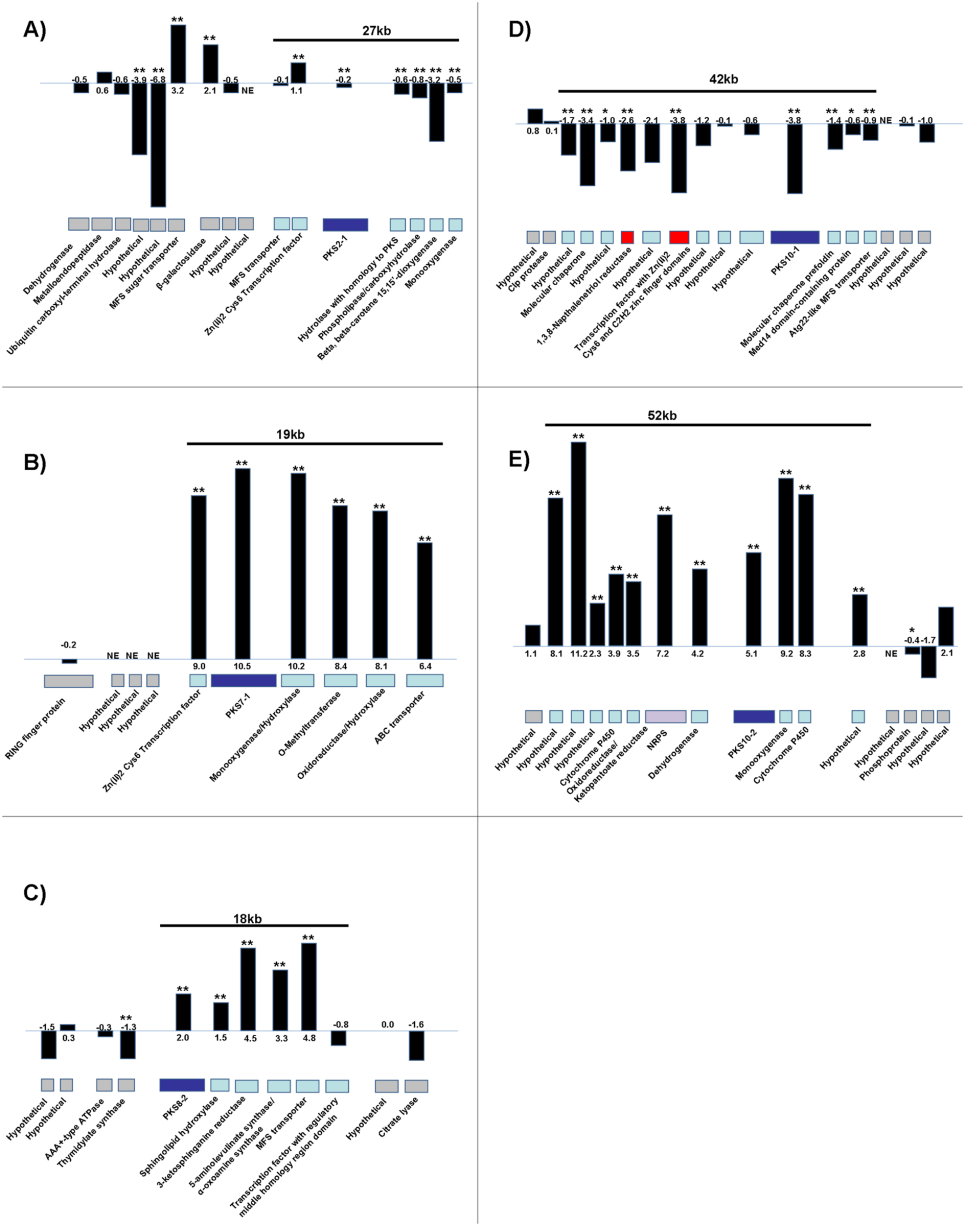
RNA-Seq results of PKS cluster gene expression in infected leaves relative to mycelial culture. Potted banana plants or PDB medium were inoculated with conidia of *M*. *fijiensis* isolate 14H1-11A, and tissue was harvested for RNA-Seq analysis. The PKS is shown, surrounded by its neighboring genes in the genome. Each gene is labeled with its function as predicted by BLAST. PKS genes are colored in dark blue, NRPS genes are colored in purple, genes known to be involved in melanin biosynthesis are colored in red, putative biosynthetic cluster genes based on a combination of bioinformatics predictions and fold changes are colored in light blue, and putative flanking genes are colored in gray. For each gene, log2 fold change values (log2FC) are shown for expression in the infected tissue versus expression in liquid culture. Expression data shown as black bars; actual log2FC values are shown at the base of the bars. Gene expression differences that are significant at p<0.05 are indicated by a single asterisk, and gene expression differences that are significant at p<0.01 are indicated by two asterisks. NE = no expression detected. A) *PKS2-1* cluster; B) *PKS7-1* cluster; C) *PKS8-2* cluster; D) *PKS10-1* (melanin) cluster; E) *PKS10-2* cluster.

We then conducted RNA-Seq analysis to further characterize expression of PKS genes as well as the other genes proposed to be in each cluster. We inoculated potted 'Grand Nain' banana plants grown in the greenhouse or PDB flasks with *M*. *fijiensis* isolate 14H1-11A. Mycelium from PDB flasks was harvested after 1 week, and infected leaves were harvested at 6 weeks post-inoculation. RNA was isolated from these tissue samples and sequenced using the Illumina HiSeq platform. We focused our analysis on the PKS genes and clusters identified as being of interest through our phylogenetic, PKS domain, and cluster analysis (Figs 4–7). Results are shown in Fig 12.

In this dataset, *PKS2-1* showed a small but statistically significant decreased expression in the infected leaf compared to when grown in PDB (Fig 12A). These results are consistent with the greater expression found from mycelial culture relative to infected tissue-cultured plants in the initial RT-PCR analysis (Fig 11). With the exception of the transcription factor, all of the genes predicted to be in the cluster (MFS transporter, hydrolase, phospholipase, dioxygenase, and monooxygenase) (Figs 3A and 5B) were also all unchanged or significantly decreased in expression in infected leaves relative to mycelial culture (Fig 12A).

In contrast to *PKS2-1*, RNA-Seq analysis showed *PKS7-1* to have a highly significant, large increase in expression in the infected leaf as compared to when grown in liquid medium (Fig 12B). All of the genes predicted to be in the cluster (transcription factor, monooxygenase, O-methyltransferase, oxidoreductase, and ABC transporter genes) (Fig 3B) showed similar increases in expression, while genes beyond those such as three hypothetical genes and a RING finger protein gene did not show such differences.

RNA-Seq analysis of *PKS8-2* showed a statistically significant increase in expression in the infected leaves relative to when grown in PDB (Fig 12C). The neighboring sphingolipid hydroxylase, ketosphinganine reductase, α-oxoamine synthase, and MFS transporter predicted to be part of the biosynthetic cluster (Figs 3D and 6B) also had statistically significant increases in expression in infected leaf tissue, whereas the neighboring transcription factor gene was not differentially expressed.

RNA-Seq analysis showed a large, statistically significant decrease in expression of *PKS10-1* in the infected leaf versus when grown in PDB (Fig 12D). Fungal melanin biosynthesis is known to require a 1,3,8-trihydroxynaphthalene reductase [51,52], and a transcription factor [53], both of which are clustered with the melanin PKS. These two genes also had decreased expression in infected leaf tissue as compared to when grown in PDB (Fig 12D). In addition to the genes known or predicted to be involved in melanin biosynthesis (Fig 3G), six more flanking genes had significantly decreased expression in infected leaf tissue: two molecular chaperone genes, a gene for a Med14-domain containing protein, two genes for hypothetical proteins, and a vacuolar transporter-like gene (Fig 12D).

Finally, RNA-Seq analysis of *PKS10-2* showed a large, statistically significant increase in expression in infected leaf tissue relative to when grown in PDB (Fig 12E). Ten neighboring genes also had large, significant increases in expression, suggesting that the cluster is larger than predicted (Figs 3H and 7B) and may include four hypothetical proteins, two cytochrome P450s, an oxidoreductase with homology to ketopantoate reductase, a non-ribosomal peptide synthase, a dehydrogenase, and a monooxygenase. The RNA-Seq results are also consistent with the RT-PCR analysis from tissue-cultured plants showing expression only in infected leaf tissue and not in culture (Fig 11).

## Discussion

While polyketide toxins have long been suspected as pathogenicity factors from *M*. *fijiensis*, this is the first study to investigate the repertoire of polyketide biosynthetic genes from this fungus. Using the publicly available genome sequence, we have predicted that there are seven PKS genes and one hybrid PKS-NRPS gene in the genome of *M*. *fijiensis*. When compared to the number of PKS gene clusters predicted from other fungi, this is relatively few (Fig 1). However, some fungal species with few PKS genes are known to produce polyketides as pathogenicity factors. For example, only four PKS genes were detected in the genome of *Dothistroma septosporum* (Fig 1, S2 Table), yet it is known to produce the polyketide dothistromin, which is an important pathogenicity factor (10). Only 6 PKS genes were detected in the genome of *Alternaria brassicicola* (Fig 1, S2 Table), yet it is known to produce the polyketide depudecin, which acts as a histone deacetylase inhibitor and contributes to virulence [54]. Therefore, it is not possible to predict the importance of polyketides in virulence based on the number of PKS genes in the genome.

All of the *M*. *fijiensis* PKS and hybrid PKS-NRPS genes were predicted to encode enzymes with all of the necessary PKS domains (KS, AT, and ACP). They contain different combinations of optional domains, which are consistent with PKS7-1, PKS8-1, and PKS10-1 being non-reducing; Hybrid8-3, PKS8-4, and PKS10-2 being partially reducing; and PKS2-1 and PKS8-2 being highly reducing PKS enzymes.

In fungal genomes, secondary metabolite biosynthetic genes are typically clustered together, and genes such as those encoding transcription factors, transporters, oxidoreductases and dehydrogenases are known to commonly be found in biosynthetic clusters [32]. Therefore, we were able to predict which genes may be part of biosynthetic clusters with each *M*. *fijiensis* PKS or hybrid PKS-NRPS (Fig 3). The sizes of these predicted biosynthetic clusters ranged from 18 to 39 kb, which is very consistent with sizes of characterized polyketide biosynthetic clusters from other fungi. For example, the bikaverin, solanapyrone, cercosporin, fumonisin, and aflatoxin clusters are, respectively 18 kb, 21 kb, 36 kb, 46 kb, and 82 kb [34,35,46,55,56,57]. Our RNA-Seq experiment provided further clues as to which genes may be co-regulated and part of a biosynthetic cluster. These data largely confirmed our bioinformatics predictions. For example, we predicted that the *PKS7-1* cluster would include a transcription factor, a mono-oxygenase, an O-methyltransferase, an oxidoreductase, and an ABC transporter (Fig 3B). The RNA-Seq data showed that all of these genes had higher expression in infected leaf tissue, with log2 fold change values of at least 6.4, whereas other neighboring genes were not differentially expressed (Fig 12B). RNA-Seq analysis also confirmed cluster predictions for the *PKS2-1* and *8–2* (Figs 3, 5B, 6B and 12). By contrast, RNA-Seq data enabled us to refine our prediction for the *PKS10-1* and *10–2* clusters: while all of the genes predicted to be in the clusters were differentially expressed, there were several neighboring hypothetical genes that had similar differential expression, suggesting that they are part of the clusters (Figs 3, 7 and 12).

Phylogenetic analysis of the *M*. *fijiensis* PKS protein sequences and well-characterized sequences from other species revealed that PKS10-1 is clearly part of the clade of melanin-producing PKS enzymes (Fig 4). *M*. *fijiensis* is highly melanized in culture, and melanin shunt metabolites such as 2,4,8-trihydroxytetralone, 4-hydroxyscytalone and juglone have been shown to be toxic to banana and have been implicated as toxins important in disease development [11,15,16,17,18]. Our expression analysis showed that the melanin *PKS10-1* is expressed in infected leaf tissue (Fig 11), however RNA-Seq analysis (Fig 12D) identified a significant down regulation of the cluster genes in leaf tissue relative to expression in culture. These results suggest that melanin shunt metabolites may not be involved in disease development, at least at the stages assayed in our studies (S1 Fig). Our expression studies do not address the possible role of melanin in other stages of pathogenicity such as entry into host tissue.

None of the other *M*. *fijiensis* PKS protein sequences had close, well-characterized homologs in our phylogenetic analysis (Fig 4). Further, protein sequences of genes in the predicted PKS clusters also had low sequence similarity to the types of genes clustered with the closest well-characterized PKS homolog (S4 and S5 Tables). These results suggest that the PKS enzymes other than PKS10-1 are likely to produce novel compounds.

In spite of the lack of clear PKS homologs, some predictions can be made based on clades in the phylogenetic analysis, PKS domain organization, and modeling of the KS domain. For example, the closest well-characterized homologs of PKS2-1 are the alternapyrone and T-toxin PKS sequences (Fig 4). While PKS2-1 has a very different biosynthetic cluster (Fig 5B) and is unlikely to produce the same product, the alternapyrone and T-toxin PKS enzymes both produce highly saturated products, which supports our prediction from the domain analysis that PKS2-1 is a highly saturating PKS (Fig 2). Furthermore, the alternapyrone and T-toxin PKS enzymes catalyze 10 and 20 iterations respectively, which is more than the other well-characterized PKS enzymes in Fig 4, and suggests that the product of PKS2-1 is also large. PKS2-1 is highly homologous (≥79% sequence similarity) with as yet uncharacterized PKS proteins within several members of the Mycosphaerellaceae including *M*. *musicola*, *M*. *eumusae*, *M*. *graminicola*, *P*. *pini-densiflorae*, and several *Zymoseptoria* species (Table 2). Furthermore, the genes predicted to be in the PKS2-1 biosynthetic clusters of *M*. *fijiensis*, *M*. *musicola*, and *M*. *eumusae* are all very similar. Our results suggest that the product may be commonly produced by this group of fungi. In our expression analysis, *PKS2-1* and its clustered genes were more highly expressed in culture than in leaf tissue (Fig 12A), suggesting a possible role in normal growth processes and not specifically in pathogenicity.

The *PKS10-2* cluster is of significant interest to us given its high expression in leaf tissue suggesting a possible role in disease development. Further, sequence similarity to PKS proteins from the related banana pathogens *M*. *musicola* and *M*. *eumusae* was >80%, whereas there was little similarity (<60%) to PKS proteins in other Mycosphaerellaceae (Table 2). Comparison of the *M*. *fijiensis PKS10-2* gene cluster with clusters from *M*. *musicola* and *M*. *eumusae* showed that the three species have nearly identical gene clusters. All of the genes predicted to be in the *M*. *fijiensis PKS10-2* gene cluster have close homologs in the other two fungi. Thus PKS10-2 may be of interest for further investigation for a possible role in pathogenicity of banana. PKS10-2 has a high bootstrap support value to a solanapyrone-producing PKS from *Alternaria solani* (Fig 4). Solanapyrone is a phytotoxic polyketide that inhibits DNA polymerase [58]. It has been thought to play a role in pathogenicity since it is less toxic to non-host than to host plants [59] although it is not essential for pathogenicity [60].

The solanapyrone and putative *PKS10-2* biosynthetic clusters also share some similar types of genes (PKS, cytochrome P450, and dehydrogenase). However, the putative *PKS10-2* cluster also contains an NRPS and other types of genes not found in the solanapyrone biosynthetic cluster (Fig 7B). Therefore, these are unlikely to produce the same compound. This conclusion is supported by the low sequence similarity between the clustered dehydrogenase and cytochrome P450 genes in the *PKS10-2* and solanapyrone clusters (S5B Table).

RNA-Seq analysis also showed the *M*. *fijiensis PKS8-2* cluster to be upregulated in leaf tissue relative to growth in culture (Fig 12C). Similarity to PKS proteins in other Mycospharellaceae was not strong (<70%) (Table 2), but PKS8-2 has a high bootstrap support value to the fumonisin-producing PKS (Fig 4). The fumonisin and *PKS8-2* biosynthetic clusters both contain genes homologous to α-oxoamine synthase (Fig 6B) [39]. α-oxoamine synthase is an enzyme involved in sphingolipid biosynthesis [61,62]. A homolog is used in fumonisin biosynthesis to produce a polyketide that can act as an analog of sphinganine and thereby inhibit subsequent steps in sphingolipid biosynthesis [39,62]. In addition to the α-oxoamine synthase homolog, the predicted *PKS8-2* cluster also includes genes homologous to ketosphinganine reductase and sphingolipid hydroxylase (Fig 6B), which further suggests that the polyketide produced by the *PKS8-2* cluster may perturb sphingolipid metabolism. Disruption of sphingolipid biosynthesis by fumonisin results in toxicity because sphingolipids are important components of eukaryotic cell membranes, and are involved in signal transduction for a variety of processes [63,64]. Fumonisin is an important pathogenicity factor, causing necrosis in sensitive plant hosts [65]. It is also cytotoxic and carcinogenic to animals [66].

Of all the clusters, the *PKS7-1* cluster remains a mystery. It is of interest due to its high expression in infected plant tissue in the RNA-Seq experiment. The genes clustered with *PKS7-1* (oxidoreductase, monooxygenase, O-methyltransferase) are also similar to genes in the biosynthetic cluster for the production of cercosporin, a light-activated toxin produced by the related *Cercospora* species that has been shown to be involved in disease development [6,55]. BLAST analysis, however, did not identify any close homologs (Fig 4, Table 2), including homologs in the banana pathogens *M*. *musicola* and *M*. *eumusae*. Thus PKS7-1 seems to be unique among PKS enzymes identified to date.

In conclusion, we have identified eight PKS or hybrid PKS-NRPS biosynthetic gene clusters in the *M*. *fijiensis* genome and we have used bioinformatics to make predictions about the products synthesized by these gene clusters. Our data predict that *PKS10-1* is involved in melanin biosynthesis, and that three other PKS clusters (*PKS2-1*, *8–2*, and *10–2*) are similar to clusters that produce alternapyrone, fumonisin, and solanapyrone, respectively. Four of the clusters (*PKS2-1*, *Hybrid8-3*, *PKS10-1*, and *PKS10-*2) are found in both of the related banana pathogens *M*. *musicola* and *M*. *eumusae* (additionally, *PKS8-4* is found in *M*. *musicola* only), however three of the clusters (*PKS7-1*, *PKS8-1*, and *PKS8-2*) are not found in these related species. Three of the clusters (*PKS7-1*, *PKS8-2*, and *PKS10-2*) are highly expressed in infected leaf tissue and are thus potential targets for further characterization of these pathways and polyketide products for a role in *M*. *fijiensis* pathogenicity.

## Methods

### Prediction of Polyketide Synthase Gene Clusters

To identify polyketide synthase gene clusters from the *M*. *fijiensis* genome as well as genomes of 74 other Dothideomycete fungi (S2 Table) [67,68], the SMURF (Secondary Metabolites Unique Region Finder) tool from J. Craig Venter Institute was used. SMURF identifies PKS and NRPS genes from user-provided protein sequences in FASTA format, and predicts which genes may be a part of the biosynthetic cluster based on genome coordinate information [32].

For each PKS or hybrid PKS-NRPS gene predicted in *M*. *fijiensis*, a blastp search was done against the non-redundant protein sequences in the NCBI database. NCBI's Conserved Domain Database [36] was used to predict the domains of each PKS or PKS-like protein sequence (Fig 2). Open reading frames near each PKS locus were identified using the JGI *M*. *fijiensis* genome browser. Blastp searches were done of each of these open reading frames to predict possible functions and compare them to types of genes commonly found in PKS gene clusters.

### Identifying Homologs of *M*. *fijiensis* PKS Genes

A phylogenetic tree was created with all of the PKS protein sequences from *M*. *fijiensis*, as well as the protein sequences of some well-characterized PKS enzymes from other species. Sequences were aligned using the MUSCLE algorithm v3.8.31 [69] in Mesquite v3.04 [70] and ModelGenerator v0.85 [71] was used to identify the best evolutionary model. The best evolutionary model was predicted by both the Akaike Information Criterion and the Bayesian Information Criterion to be LG+I+G+F [72]. Therefore, this model was used with RaxmlGUI v1.3.1 [73] to generate the phylogenetic tree, using maximum likelihood with slow bootstrap, no outgroup, and the autoMRE function.

Repeat masked assembly scaffolds were downloaded for 93 Dothideomycete fungal genomes available on JGI, as well as 10 additional Mycosphaerellaceae genomes available on NCBI (S7 Table). BLAST+ [74] was used to create a local blast database of these Dothideomycete genome sequences, and this database was searched for hits of the *M*. *fijiensis* PKS sequences, using the tblastn algorithm. Results were sorted by bitscore and color coded by percent sequence similarity (Table 2).

### Ketosynthase Domain Alignment and Prediction of Iteration Number

KS domain sequences were identified using the University of Maryland PKS/NRPS Analysis Web Server [50], for each of the *M*. *fijiensis* PKS protein sequences and the other PKS protein sequences used for creating the RAxML phylogeny. These were aligned using MUSCLE v3.8.31 in Mesquite v3.04, along with the 1kas fatty acid synthesis enzyme sequence (Accession 13BN_A). Amino acid residues corresponding to positions 229 and 400 in 1kas were determined for each sequence.

### Fungal Cultures

Isolate 10CR1-24 was obtained from infected banana leaves collected from a commercial banana plantation in Guapiles, Costa Rica. Single ascospore isolations were done based on the protocol kindly provided by Dr. Miguel Muñoz, Dole Food Company (Personal Communication). Regions of infected leaves containing pseudothecia were cut into 2 cm squares, stapled to paper and submerged in sterile water for 10 minutes at room temperature to allow pseudothecia to hydrate. Leaf squares were attached to the lids of petri dishes suspended above 1% water agar, and incubated for 60 minutes at room temperature to allow ascospores to be released. Presence of ascospores on the water agar surface was confirmed using a dissecting microscope, and ascospores were recovered by pipetting into sterile water. The resulting spore suspension was transferred to a new Potato Dextrose Agar (PDA) (BD Difco) or Mycophil agar (BD Difco) plate, and a cell spreader was used to spread the ascospores across the plate. Plates were sealed with parafilm and incubated at 25°C to allow colonies to grow. Isolate 14H1-11A was obtained using the same method and was kindly provided by Jean Ristaino (North Carolina State University). The species of each resulting colony was confirmed by ITS sequencing followed by BLAST: ITS sequences of 10CR1-24 and 14H1-11A have 100% identity with *M*. *fijiensis* isolate UQ H444 (Accession AY923762.1).

Conidia were obtained from isolates using the protocol of Peraza-Echeverria et al [75]. Briefly, mycelial cultures of *M*. *fijiensis* isolates 10CR1-24 and 14H1-11A grown on PDA medium were macerated in water, and 2 mL of the resulting suspension was pipetted onto plates of modified V8 medium (0.2g/L CaCO_3_, 100 mL/L V8 juice and 20g/L Difco agar). Cultures were incubated at 18°C under continuous, cool-white fluorescent and black light. After 5–6 days, sporulation plates were stimulated to produce conidia by adding 2 mL water and brushing the plates with a paint brush or cell spreader, and removing the resulting suspension. After another 5–6 days, conidia were harvested in the same way, adding 2 mL water or 0.5% Tween 20 solution, brushing the plates to dislodge spores, and removing the spore suspension by pipetting.

### Banana Tissue Culture and Inoculation

Grand Nain banana tissue culture plants (kindly provided by Miguel Muñoz, Dole Food Company) were maintained on modified Murashige and Skoog medium [76]. Growth medium was prepared with 4.33 g/L Murashige and Skoog basal salts (Caisson labs), 30g/L sucrose, 200 ug/L glycine, 50 ug/L niacin, 50 ug/L pyridoxine, 10 ug/L thiamin, 10 ug/L myo-inositol, 1 ug/L cysteine, 2 g/L Phytagel (Sigma-Aldrich), with or without 4.5 mg/L 6-benzylaminopurine (BAP), with the pH adjusted to 5.8. Medium with BAP is used for bud proliferation, while medium without BAP is used for rooting. Plants were maintained on an 18h light/6h dark photoperiod with cool white fluorescent light at 25–30°C.

### Growth Conditions for Semi-Quantitative RT-PCR and RNA-Seq Assays

For semi-quantitative RT-PCR assays, in vitro-cultured banana plants grown on medium without BAP were used for inoculations. A mix of conidia and mycelial fragments harvested from V8 sporulation plates of isolate 10CR1-24 described above were applied as 10 μL droplets onto the leaves. Plants were maintained under an 18h light/6h dark photoperiod before harvesting lesions at 5 weeks post-inoculation by cutting out the lesions and flash-freezing them in liquid nitrogen. For growth in culture, macerated mycelium of 10CR1-24 harvested from PDA plates was grown in 100 mL of PDB in 250 mL flasks. Flasks were incubated at 28°C in an incubator shaker at 250 rpm for 1 week in the dark. Tissue was harvested by filtering through Miracloth (Millipore), blotting the tissue dry, and flash-freezing in liquid nitrogen. For both infected leaf tissue and mycelial tissue, tissue was ground using a mortar and pestle, RNA was extracted using the Spectrum Plant Total RNA kit, and samples were DNase treated with TURBO DNase (Ambion). RNA quality was verified by gel electrophoresis with 300 ng RNA as estimated by Nanodrop (Thermo Scientific). cDNA was synthesized using iScript Select cDNA synthesis kit (Bio-Rad). PCR reactions used ExTaq (TaKaRa) according to manufacturer's instructions, with primers, annealing temperatures, extension times, and cycle numbers as indicated in S10 Table.

For RNA-Seq analysis, rooted in vitro-cultured banana plants were transferred to soil and grown in the greenhouse until plants were approximately 20 cm in height, after which they were moved to an incubator at 25°C and under an18h light/6h dark photoperiod, with cool white light. Conidia of isolate 14H1-11A were diluted in sterile 0.5% Tween 20 to a final concentration of 5.2x10^4^/mL, and 25 mL of the conidia suspension was sprayed onto each plant. For the first week post-inoculation, plants were covered in clear plastic bags to maintain high humidity conditions conducive to infection. After 6 weeks post-inoculation, symptomatic leaves were harvested by cutting out leaf areas with lesions, and flash freezing in liquid nitrogen. For mycelial cultures for RNA-Seq, 10 μL of 1.3x10^6^/mL conidia of 14H1-11A were used to inoculate 50 mL PDB in 125 mL flasks. Flasks were incubated on a rotary shaker at 150 rpm at 25°C in the dark. After 1 week, mycelium was harvested by filtering through Miracloth and flash freezing in liquid nitrogen. RNA was extracted from the infected leaf and mycelial tissue and purity was confirmed as described above for the semi-quantitative RT-PCR experiment except that RNA samples were DNAse treated using DNase I (Roche).

### cDNA Library Construction and Illumina HiSeq Sequencing

Total RNA was submitted to the North Carolina State University Genomic Sciences Laboratory for sequencing. Quality of RNA was further confirmed using an Agilent Bioanalyzer, and then strand-specific libraries were created using the NEBNext Ultra Directional library prep kit (New England BioLabs). Sequencing was done using an Illumina HiSeq 2500 platform to generate 125-base single-end reads. Sequencing resulted in an average yield of 32 million reads per sample. RNA-Seq data are available through NCBI through SRP075820.

### Identification of Differentially Expressed Genes

FastQC (http://www.bioinformatics.babraham.ac.uk/projects/fastqc/) was used to verify quality of the RNA-Seq reads for each sample. Illumina Truseq adapter sequences and low-quality bases were trimmed using CutAdapt v1.7 with a quality cutoff of 20 and a minimum sequence length of 36 [77].

Sequences from each sample were mapped to the banana genome, *Musa acuminata* subsp. *malaccensis* double-haploid Pahang [78], and to the *M*. *fijiensis* genome [27] using Tophat v2.0.9 [79]. Gene expression levels were determined using HTSeq v0.6.0 [80], with gene annotations available from JGI. DESeq2 v1.4.5 was used to identify differentially expressed genes [81].

## Supporting Information

S1 Fig Examples of black Sigatoka symptoms. A) Infected leaf on banana plant in Costa Rica B) Infected banana plants in Costa Rica banana plantation C) Example of infected tissue culture plant D) Example of infected leaf harvested for transcriptome analysis, adaxial side E) Abaxial side.
(TIF)

S1 Table Accession numbers for PKS sequences. Accession numbers or JGI protein ID numbers for *M*. *fijiensis* PKS protein sequences, and well-characterized PKS protein sequences from other species. Names of PKS proteins are indicated as well as the polyketide product produced.
(DOC)

S2 Table Number of PKS genes identified in genomes of Dothideomycete fungi. For each species, the number of PKS genes identified by SMURF is indicated along with the name of each species' taxonomic order.
(DOC)

S3 Table E-values for domains in each *M*. *fijiensis* PKS or hybrid enzyme. Blastp with the Conserved Domain Database from NCBI was used to predict enzyme domains in each PKS or PKS-NRPS enzyme. E-values are shown for each domain predicted. A) *M*. *fijiensis* PKS enzymes. B) *M*. *fijiensis* hybrid PKS-NRPS enzyme. Abbreviations for domains: SAT = starter unit acyltransferase; KS = ketosynthase; AT = acyltransferase; PT = product template; DH = dehydratase; MT = methyltransferase; ER = enoyl reductase; KR = ketoreductase; ACP 1 = first acyl carrier protein domain; ACP 2 = second acyl carrier protein domain; TE = thioesterase; C = condensation; Hx = HxxPF repeat domain; A = adenylation. The Conserved Domain Database also identifies binding sites. Shown are the presence (NAD(P)+) or absence (NAD(P)-) of an NAD(P) binding site in the ER, KR, and TE domains; presence (SAM+) or absence (SAM-) of a SAM binding site in the MT domain; and presence of an AMP binding site (AMP +) and acyl-activating enzyme consensus motif (Acyl-act+) in the A domain. Some PKS proteins such as those for melanin and cercosporin (CTB1) are known to have a SAT domain at the N terminus. Blastp searches were done against each *M*. *fijiensis* PKS or hybrid PKS-NRPS, using the region containing the SAT domain in CTB1. E-values for hits for this search are shown in red text.
(DOC)

S4 Table Blast hits for putative *M*. *fijiensis* polyketide biosynthetic cluster gene. For each polyketide biosynthetic cluster with its neighboring genes in *M*. *fijiensis*, the table shows the description of the gene in Figs 3 and 12, the transcript name and protein ID for JGI, the accession number with a link to the relevant page on NCBI, the position of the gene on the scaffold with a link to the genome browser on JGI, and the gene annotations available through JGI (GO description, Interpro description, and KOG description). Blastp analysis was done for each gene using the non-redundant protein sequence database on NCBI, and the top ten hits are indicated with species where they are found, description of the gene, bitscore, E-value, percent sequence identity and similarity, and accession (with a link to the NCBI page). Domains identified by the Conserved Domain Database are also indicated, with their associated E-values.
(XLS)

S5 Table Sequence similarity for proteins with similar functions encoded by polyketide biosynthetic clusters. A) *M*. *fijiensis* PKS8-2 cluster compared to fumonisin biosynthetic cluster from *F*. *verticillioides*; B) *M*. *fijiensis* PKS10-2 cluster compared to solanapyrone biosynthetic cluster from *A*. *solani*.
(DOC)

S6 Table Amino acid residues for tertiary structure analysis of ketosynthase domains. Amino acid residues for *M*. *fijiensis* PKS sequences and sequences from well-characterized PKS proteins, corresponding to positions 229 and 400 in the fatty acid synthase 1kas. For well-characterized PKS enzymes, the number of iterations catalyzed is shown. Also indicated is whether each PKS is more closely related to the PKS producing 6-methylsalicylic acid (MSAS), naphthopyrone (NAP), or T-toxin (T-tox) (Fig 4), and the number of iterations catalyzed by members of this clade, if known. These are color coded yellow, blue, or pink, respectively. For *M*. *fijiensis* PKS sequences in the T-toxin PKS clade, well-characterized PKS enzymes with identical residues at positions 229 and 400 are indicated, along with the number of iterations catalyzed by those PKS enzymes.
(DOC)

S7 Table List of Dothideomycete fungal genomes used to identify *M*. *fijiensis* PKS homologs. Dothideomycete fungal genomes from JGI and NCBI, for tblastn searches for each *M*. *fijiensis* PKS sequence, to identify uncharacterized homologs of each PKS.
(DOC)

S8 Table Conserved domains in *PKS2-1*, *PKS8-4*, and *PKS10-2* gene clusters across *Mycosphaerella* species. For the *M*. *fijiensis PKS2-1*, *PKS8-4*, and *PKS10-2* gene clusters, orthologous gene clusters were identified from *M*. *musicola* and *M*. *eumusae*. Conserved domains were predicted from each protein encoded by genes in the *M*. *musicola* or *M*. *eumusae* gene clusters. The table indicates the NCBI accession number for each predicted protein, the JGI protein ID, the location of the gene on the scaffold, the description of the protein in Figs 8–10, and the conserved domains of each protein as predicted by the Conserved Domain Database. Each tab shows the results for a different biosynthetic cluster. A) *PKS2-1* orthologous cluster in *M*. *musicola*; B) *PKS2-1* orthologous cluster in *M*. *eumusae*; C) *PKS8-4* orthologous cluster in *M*. *musicola*; D) *PKS10-2* orthologous cluster in *M*. *musicola*; E) *PKS10-2* orthologous cluster in *M*. *eumusae*.
(XLS)

S9 Table Percent identity and similarity for orthologs of *M*. *fijiensis* polyketide biosynthesis proteins. For each putative polyketide biosynthetic protein sequence from *M*. *fijiensis*, the table indicates the ortholog of the sequence in *M*. *musicola* or *M*. *eumusae*. NCBI accession numbers are shown for the *M*. *fijiensis* sequence and its ortholog, as well as the percent identity and similarity of the sequences. Each spreadsheet tab shows the results for a different polyketide biosynthetic cluster. A) *PKS2-1* orthologous cluster in *M*. *musicola*; B) *PKS2-1* orthologous cluster in *M*. *eumusae*; C) *PKS8-4* orthologous cluster in *M*. *musicola*; D) *PKS10-2* orthologous cluster in *M*. *musicola*; E) *PKS10-2* orthologous cluster in *M*. *eumusae*.
(XLS)

S10 Table Primer sets and conditions for semi-quantitative RT-PCR assays. For each RT-PCR assay, primer names, sequences, annealing temperature, extension time, number of cycles, and expected product sizes are indicated. * Same product size expected for cDNA and gDNA products.
(DOC)
