# Supplementary figures and images for: Bioinformatics Prediction of Polyketide Synthase Gene Clusters from Mycosphaerella fijiensis
Source: PLoS One. 2016 Jul 7;11(7):e0158471. doi: 10.1371/journal.pone.0158471 (PMC4936691; doi:10.1371/journal.pone.0158471)

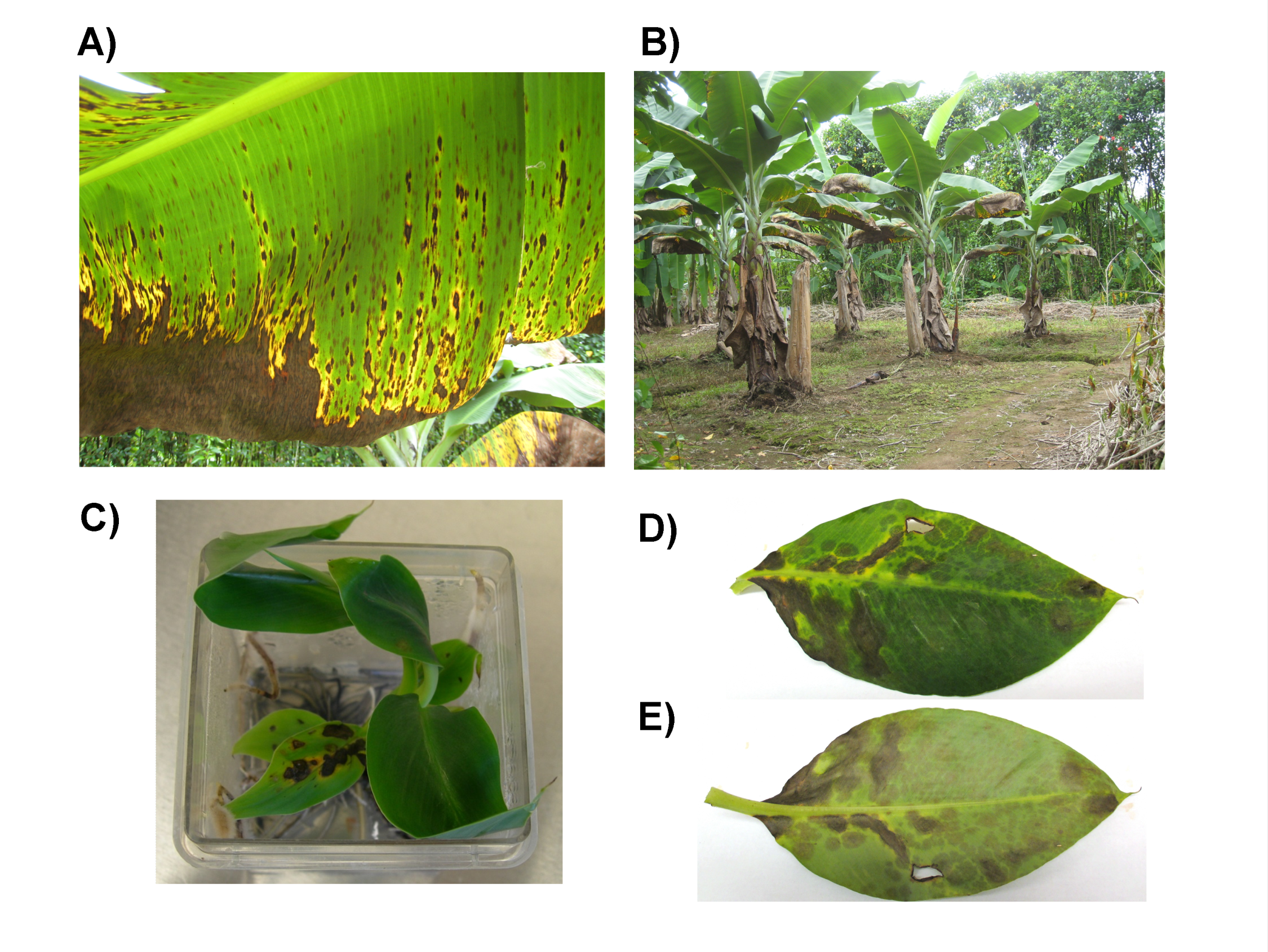

Supplement: S1 Fig — A) Infected leaf on banana plant in Costa Rica B) Infected banana plants in Costa Rica banana plantation C) Example of infected tissue culture plant D) Example of infected leaf harvested for transcriptome analysis, adaxial side E) Abaxial side. (TIF) [file pone.0158471.s001.tif]
