# Supplementary material for: Bioinformatics Prediction of Polyketide Synthase Gene Clusters from Mycosphaerella fijiensis
Source: PLoS One. 2016 Jul 7;11(7):e0158471. doi: 10.1371/journal.pone.0158471 (PMC4936691; doi:10.1371/journal.pone.0158471)
Supplement: S1 Table — Accession numbers or JGI protein ID numbers for M. fijiensis PKS protein sequences, and well-characterized PKS protein sequences from other species. Names of PKS proteins are indicated as well as the polyketide product produced. (DOC) [file pone.0158471.s002.doc]

**S1 Table.** **Accession numbers for PKS sequences.** Accession numbers or JGI protein ID numbers for *M. fijiensis* PKS protein sequences, and well-characterized PKS protein sequences from other species. Names of PKS proteins are indicated as well as the polyketide product produced.

| **Species** | **Polyketide product** | **Protein name** | **Citation** | **Accession number** | **JGI protein ID** |
| --- | --- | --- | --- | --- | --- |
| *Mycosphaerella fijiensis* | - | PKS2-1 |  | XP_007922357.1 | 29867 |
| *Mycosphaerella fijiensis* | - | PKS7-1 |  | XP_007928748.1 (partial sequence) | 141883 (full sequence) |
| *Mycosphaerella fijiensis* | - | PKS8-1 |  | XP_007929626.1 | 34361 |
| *Mycosphaerella fijiensis* | - | PKS8-2 |  | XP_007929903.1 | 166988 |
| *Mycosphaerella fijiensis* | - | Hybrid8-3 |  | XP_007930181.1 | 93879 |
| *Mycosphaerella fijiensis* | - | PKS8-4 |  | XP_007930394.1 | 190533 |
| *Mycosphaerella fijiensis* | - | PKS10-1 |  | XP_007931490.1 | 216850 |
| *Mycosphaerella fijiensis* | - | PKS10-2 |  | XP_007931278.1 | 44966 |
| *Bipolaris oryzae* | Melanin | PKS1 |  | BAD22832.1 | - |
| *Colletotrichum graminicola* | Melanin | PKS1 |  | ACN32207.2 | - |
| *Exophiala dermatitidis* | Melanin | PKS1 |  | AAD31436.3 | - |
| *Glarea lozoyensis* | Melanin | PKS1 |  | AAN59953.1 | - |
| *Cercospora nicotianae* | Cercosporin | CTB1 |  | AAT69682.1 | - |
| *Cercospora zeae-maydis* | Cercosporin | CTB1 |  | - | 42949 |
| *Aspergillus parasiticus* | Aflatoxin | PKSL1 |  | Q12053.1 | - |
| *Fusarium fujikuroi* | Bikaverin | PKS4 |  | CCT67991.1 | - |
| *Penicillium citrinum* | Compactin | MlcA |  | BAC20564.1 | - |
| *Aspergillus terreus* | Lovastatin | LNKS |  | Q9Y8A5.1 | - |
| *Aspergillus nidulans* | Sterigmatocystin | PKSst |  | XP_681094.1 | - |
| *Fusarium verticillioides* | Fumonisin | Fum1p |  | AAD43562.2 | - |
| *Alternaria solani* | Alternapyrone | PKSN |  | BAD83684.1 | - |
| *Alternaria solani* | Solanapyrone | Sol1 |  | BAJ09789.1 | - |
| *Bipolaris maydis* | T-toxin | PKS1 |  | AAB08104.3 | - |
| *Penicillium griseofulvum* | 6-methylsalicylic acid | MSAS |  | CAA39295.1 | - |
| *Aspergillus nidulans* | naphthopyrone YWA1 | WA |  | CAA46695.2 | - |

**Literature Cited**

1. Moriwaki A, Kihara J, Kobayashi T, Tokunaga T, Arase S, Honda Y. Insertional mutagenesis and characterization of a polyketide synthase gene (PKS1) required for melanin biosynthesis in *Bipolaris oryzae*. Fems Microbiology Letters. 2004 Sep;238(1):1-8.

2. Ludwig N, Lohrer M, Hempel M, Mathea S, Schliebner I, Menzel M, et al. Melanin is not required for turgor generation but enhances cell-wall rigidity in appressoria of the corn pathogen *Colletotrichum graminicola*. Molecular Plant-Microbe Interactions. 2014 Apr;27(4):315-27.

3. Feng B, Wang X, Hauser M, Kaufmann S, Jentsch S, Haase G, et al. Molecular cloning and characterization of WdPKS1, a gene involved in dihydroxynaphthalene melanin biosynthesis and virulence in *Wangiella* (*Exophiala*) *dermatitidis*. Infection and Immunity. 2001 Mar;69(3):1781-94.

4. Zhang A, Lu P, Dahl-Roshak AM, Paress PS, Kennedy S, Tkacz JS, et al. Efficient disruption of a polyketide synthase gene (pks1) required for melanin synthesis through *Agrobacterium*-mediated transformation of *Glarea lozoyensis*. Molecular Genetics and Genomics. 2003 Feb;268(5):645-55.

5. Choquer M, Dekkers KL, Chen HQ, Cao LH, Ueng PP, Daub ME, et al. The CTB1 gene encoding a fungal polyketide synthase is required for cercosporin biosynthesis and fungal virulence of *Cercospora nicotianae*. Molecular Plant-Microbe Interactions. 2005 May;18(5):468-76.

6. Feng GH, Leonard TJ. Characterization of the polyketide synthase gene (pksL1) required for aflatoxin biosynthesis in *Aspergillus parasiticus*. Journal of Bacteriology. 1995 Nov;177(21):6246-54.

7. Arndt B, Studt L, Wiemann P, Osmanov H, Kleigrewe K, Kohler J, et al. Genetic engineering, high resolution mass spectrometry and nuclear magnetic resonance spectroscopy elucidate the bikaverin biosynthetic pathway in *Fusarium fujikuroi*. Fungal Genetics and Biology. 2015 Nov;84:26-36.

8. Abe Y, Suzuki T, Ono C, Iwamoto K, Hosobuchi M, Yoshikawa H. Molecular cloning and characterization of an ML-236B (compactin) biosynthetic gene cluster in *Penicillium citrinum*. Molecular Genetics and Genomics. 2002 Jul;267(5):636-46.

9. Hendrickson L, Ray Davis C, Roach C, Kim Nguyen D, Aldrich T, McAda PC, et al. Lovastatin biosynthesis in *Aspergillus terreus*: Characterization of blocked mutants, enzyme activities and a multifunctional polyketide synthase gene. Chemistry & Biology. 1999;6(7):429-39.

10. Galagan JE, Calvo SE, Cuomo C, Ma LJ, Wortman JR, Batzoglou S, et al. Sequencing of *Aspergillus nidulans* and comparative analysis with *A. fumigatus* and *A. oryzae*. Nature. 2005 Dec;438(7071):1105-15.

11. Seo JA, Proctor RH, Plattner RD. Characterization of four clustered and coregulated genes associated with fumonisin biosynthesis in *Fusarium verticillioides*. Fungal Genetics and Biology. 2001 Dec;34(3):155-65.

12. Fujii I, Yoshida N, Shimomaki S, Oikawa H, Ebizuka Y. An iterative type I polyketide synthase PKSN catalyzes synthesis of the decaketide alternapyrone with regio-specific octa-methylation. Chemistry & Biology. 2005 Dec;12(12):1301-9.

13. Kasahara K, Miyamoto T, Fujimoto T, Oguri H, Tokiwano T, Oikawa H, et al. Solanapyrone synthase, a possible Diels-Alderase and iterative type I polyketide synthase encoded in a biosynthetic gene cluster from *Alternaria solani*. Chembiochem. 2010 Jun;11(9):1245-52.

14. Baker SE, Kroken S, Inderbitzin P, Asvarak T, Li BY, Shi L, et al. Two polyketide synthase-encoding genes are required for biosynthesis of the polyketide virulence factor, T-toxin, by *Cochliobolus heterostrophus*. Molecular Plant-Microbe Interactions. 2006 Feb;19(2):139-49.

15. Beck J, Ripka S, Siegner A, Schiltz E, Schweizer E. The multifunctional 6-methylsalicylic acid synthase gene of *Penicillium patulum* - its gene structure relative to that of other polyketide synthases. European Journal of Biochemistry. 1990 Sep;192(2):487-98.

16. Mayorga ME, Timberlake WE. The developmentally regulated *Aspergillus nidulans wA* gene encodes a polypeptide homologous to polyketide and fatty acid synthases. Molecular & General Genetics. 1992 Nov;235(2-3):205-12.
