# Supplementary material for: Bioinformatics Prediction of Polyketide Synthase Gene Clusters from Mycosphaerella fijiensis
Source: PLoS One. 2016 Jul 7;11(7):e0158471. doi: 10.1371/journal.pone.0158471 (PMC4936691; doi:10.1371/journal.pone.0158471)
Supplement: S2 Table — For each species, the number of PKS genes identified by SMURF is indicated along with the name of each species' taxonomic order. (DOC) [file pone.0158471.s003.doc]

**S2 Table.** **Number of PKS genes identified in genomes of Dothideomycete fungi.** For each species, the number of PKS genes identified by SMURF is indicated along with the name of each species' taxonomic order.

| **Family** | **Species** | **Number of PKS genes predicted** |
| --- | --- | --- |
| Asterinales | *Aulographum hederae* | 5 |
| Botryosphaeriales | *Saccharata proteae* | 3 |
| Botryosphaeriales | *Neofusicoccum parvum* | 15 |
| Botryosphaeriales | *Macrophomina phaseolina* | 18 |
| Botryosphaeriales | *Aplosporella prunicola* | 26 |
| Botryosphaeriales | *Botryosphaeria dothidea* | 26 |
| Capnodiales | *Acidomyces richmondensis* | 2 |
| Capnodiales | *Baudoinia compniacensis* | 2 |
| Capnodiales | *Piedraia hortae* | 2 |
| Capnodiales | *Dothistroma septosporum* | 4 |
| Capnodiales | *Teratosphaeria nubilosa* | 6 |
| Capnodiales | *Cladosporium fulvum* | 7 |
| Capnodiales | *Mycosphaerella fijiensis* | 7 |
| Capnodiales | *Mycosphaerella musicola* | 7 |
| Capnodiales | *Zymoseptoria ardabiliae* | 7 |
| Capnodiales | *Polychaeton citri* | 8 |
| Capnodiales | *Mycosphaerella eumusae* | 9 |
| Capnodiales | *Dissoconium aciculare* | 10 |
| Capnodiales | *Septoria populicola* | 10 |
| Capnodiales | *Zymoseptoria pseudotritici* | 10 |
| Capnodiales | *Cercospora zeae-maydis* | 11 |
| Capnodiales | *Mycosphaerella graminicola* | 12 |
| Capnodiales | *Pseudovirgaria hyperparasitica* | 18 |
| Capnodiales | *Septoria musiva* | 19 |
| Capnodiales | *Zasmidium cellare* | 22 |
| Dothideales | *Aureobasidium pullulans var. melanogenum* | 4 |
| Dothideales | *Aureobasidium pullulans var. namibiae* | 4 |
| Dothideales | *Aureobasidium pullulans var. pullulans* | 6 |
| Dothideales | *Aureobasidium pullulans var. subglaciale* | 11 |
| Hysteriales | *Hysterium pulicare* | 23 |
| Microthyriales | *Tothia fuscella* | 6 |
| Myriangiales | *Myriangium duriaei* | 9 |
| Mytilinidiales | *Lophium mytilinum* | 7 |
| Mytilinidiales | *Cenococcum geophilum* | 10 |
| Patellariales | *Patellaria atrata* | 8 |
| Pleosporales | *Trichodelitschia bisporula* | 1 |
| Pleosporales | *Alternaria brassicicola* | 6 |
| Pleosporales | *Macroventuria anomochaeta* | 7 |
| Pleosporales | *Sporormia fimetaria* | 7 |
| Pleosporales | *Didymella exigua* | 8 |
| Pleosporales | *Clathrospora elynae* | 10 |
| Pleosporales | *Alternaria alternata* | 11 |
| Pleosporales | *Ophiobolus disseminans* | 11 |
| Pleosporales | *Cucurbitaria berberidis* | 12 |
| Pleosporales | *Leptosphaeria maculans* | 12 |
| Pleosporales | *Pleomassaria siparia* | 12 |
| Pleosporales | *Stagonospora nodorum SN15* | 12 |
| Pleosporales | *Delitschia confertaspora* | 13 |
| Pleosporales | *Dothidotthia symphoricarpi* | 14 |
| Pleosporales | *Massarina eburnea* | 14 |
| Pleosporales | *Cochliobolus lunatus* | 15 |
| Pleosporales | *Karstenula rhodostoma* | 15 |
| Pleosporales | *Phoma tracheiphila* | 15 |
| Pleosporales | *Polyplosphaeria fusca* | 16 |
| Pleosporales | *Westerdykella ornata* | 16 |
| Pleosporales | *Cochliobolus sativus* | 17 |
| Pleosporales | *Stagonospora sp. SRC1lsM3a* | 17 |
| Pleosporales | *Verruculina enalia* | 17 |
| Pleosporales | *Cochliobolus victoriae* | 18 |
| Pleosporales | *Trematosphaeria pertusa* | 18 |
| Pleosporales | *Aaosphaeria arxii* | 19 |
| Pleosporales | *Amniculicola lignicola* | 19 |
| Pleosporales | *Lophiostoma macrostomum* | 19 |
| Pleosporales | *Pyrenophora teres f. teres* | 20 |
| Pleosporales | *Cochliobolus heterostrophus C4* | 23 |
| Pleosporales | *Setosphaeria turcica* | 23 |
| Pleosporales | *Zopfia rhizophila* | 27 |
| Pleosporales | *Lentithecium fluviatile* | 28 |
| Pleosporales | *Byssothecium circinans* | 29 |
| Pleosporales | *Corynespora cassiicola* | 29 |
| Pleosporales | *Lindgomyces ingoldianus* | 31 |
| Pleosporales | *Cochliobolus heterostrophus C5* | 33 |
| Pleosporales | *Melanomma pulvis-pyrius* | 33 |
| Trypetheliales | *Trypethelium eluteriae* | 37 |
| Venturiales | *Venturia inaequalis* | 6 |
