# Supplementary material for: Bioinformatics Prediction of Polyketide Synthase Gene Clusters from Mycosphaerella fijiensis
Source: PLoS One. 2016 Jul 7;11(7):e0158471. doi: 10.1371/journal.pone.0158471 (PMC4936691; doi:10.1371/journal.pone.0158471)
Supplement: S3 Table — Blastp with the Conserved Domain Database from NCBI was used to predict enzyme domains in each PKS or PKS-NRPS enzyme. E-values are shown for each domain predicted. A) M. fijiensis PKS enzymes. B) M. fijiensis hybrid PKS-NRPS enzyme. Abbreviations for domains: SAT = starter unit acyltransferase; KS = ketosynthase; AT = acyltransferase; PT = product template; DH = dehydratase; MT = methyltransferase; ER = enoyl reductase; KR = ketoreductase; ACP 1 = first acyl carrier protein domain; ACP 2 = second acyl carrier protein domain; TE = thioesterase; C = condensation; Hx = HxxPF repeat domain; A = adenylation. The Conserved Domain Database also identifies binding sites. Shown are the presence (NAD(P)+) or absence (NAD(P)-) of an NAD(P) binding site in the ER, KR, and TE domains; presence (SAM+) or absence (SAM-) of a SAM binding site in the MT domain; and presence of an AMP binding site (AMP +) and acyl-activating enzyme consensus motif (Acyl-act+) in the A domain. Some PKS proteins such as those for melanin and cercosporin (CTB1) are known to have a SAT domain at the N terminus. Blastp searches were done against each M. fijiensis PKS or hybrid PKS-NRPS, using the region containing the SAT domain in CTB1. E-values for hits for this search are shown in red text. (DOC) [file pone.0158471.s004.doc]

**S3 Table. E-values for domains in each *M. fijiensis* PKS or hybrid enzyme.** Blastp with the Conserved Domain Database from NCBI was used to predict enzyme domains in each PKS or PKS-NRPS enzyme. E-values are shown for each domain predicted**.** A) *M. fijiensis* PKS enzymes. B) *M. fijiensis* hybrid PKS-NRPS enzyme. Abbreviations for domains: SAT = starter unit acyltransferase; KS = ketosynthase; AT = acyltransferase; PT = product template; DH = dehydratase; MT = methyltransferase; ER = enoyl reductase; KR = ketoreductase; ACP 1 = first acyl carrier protein domain; ACP 2 = second acyl carrier protein domain; TE = thioesterase; C = condensation; Hx = HxxPF repeat domain; A = adenylation. The Conserved Domain Database also identifies binding sites. Shown are the presence (NAD(P)+) or absence (NAD(P)-) of an NAD(P) binding site in the ER, KR, and TE domains; presence (SAM+) or absence (SAM-) of a SAM binding site in the MT domain; and presence of an AMP binding site (AMP +) and acyl-activating enzyme consensus motif (Acyl-act+) in the A domain. Some PKS proteins such as those for melanin and cercosporin (CTB1) are known to have a SAT domain at the N terminus. Blastp searches were done against each *M. fijiensis* PKS or hybrid PKS-NRPS, using the region containing the SAT domain in CTB1. E-values for hits for this search are shown in red text.

A)

|  | SAT | KS | AT | PT | DH | MT | ER | KR | ACP  1 | ACP  2 | TE |
| --- | --- | --- | --- | --- | --- | --- | --- | --- | --- | --- | --- |
| PKS2-1 | N/A | 1.27e-138  Active site+ | 1.30e-102 | N/A | 5.53e-42 | N/A | 3.81e-36  NAD(P) - | 4.99e-66  Active site+  NAD(P) + | 6.76e-07 | N/A | N/A |
| PKS7-1 | N/A | 3.27e-110  Active site+ | 3.85e-62 | 4.21e-108 | N/A | N/A | N/A | N/A | 4.30e-11 | 3.26e-06 | 4.26e-22  Active site - |
| PKS8-1 | 7e-16 | 1.71e-117 Active site+ | 2.57e-96 | 1.11e-123 | N/A | N/A | N/A | N/A | 6.72e-11 | N/A | N/A |
| PKS8-2 | N/A | 6.14e-124 Active site+ | 1.56e-88 | N/A | 7.93e-58 | 2.91e-04  SAM + | 1.33e-140  NAD(P) + | 7.09e-58 | 4.38e-06 | N/A | N/A |
| PKS8-4 | N/A | 2.75e-140 Active site+ | 1.16e-82 | N/A | 8.47e-37 | 1.79e-07  SAM + | N/A | 7.41e-52  Active site +  NAD(P) + | 6.5e-04 | N/A | 4.86e-24  Active site + |
| PKS10-1 | 1.41e-04  3e-29 | 2.56e-119  Active site+ | 1.18e-97 | 6.26e-136 | N/A | N/A | N/A | N/A | 9.33e-11 | 1.35e-13 | 1.75e-23 |
| PKS10-2 | N/A | 2.56e-119  Active site+ | 1.64e-98 | N/A | 1.57e-49 | 5.42e-08  SAM + | 2.22e-05  NAD(P) - | 2.63e-56  Active site +  NAD(P) - | 9.69e-10 | N/A | N/A |

B)

|  | KS | AT | DH | MT | KR | ACP  1 | C | Hx | A | ACP  2 | TE |
| --- | --- | --- | --- | --- | --- | --- | --- | --- | --- | --- | --- |
| Hybrid8-3 | 2.32e-141  Active site+ | 1.18e-106 | 5.23e-44 | 9.99e-04  SAM + | 1.86e-49  Active site -  NAD(P) - | 8.98e-08 | 2.85e-43 | 3.8e-10 | 3.49e-112  AMP +  Acyl-act + | 8.54e-06 | 6.45e-34  Active site +  NAD(P) + |

0000000000000000000000000000000000000000000000000000000000000000000000000000000000000000000000000000000000000000000000000000000
