# Supplementary material for: Bioinformatics Prediction of Polyketide Synthase Gene Clusters from Mycosphaerella fijiensis
Source: PLoS One. 2016 Jul 7;11(7):e0158471. doi: 10.1371/journal.pone.0158471 (PMC4936691; doi:10.1371/journal.pone.0158471)
Supplement: S5 Table — A) M. fijiensis PKS8-2 cluster compared to fumonisin biosynthetic cluster from F. verticillioides; B) M. fijiensis PKS10-2 cluster compared to solanapyrone biosynthetic cluster from A. solani. (DOC) [file pone.0158471.s006.doc]

**S5 Table.** **Sequence similarity for proteins with similar functions encoded by polyketide biosynthetic clusters.** A) *M. fijiensis* PKS8-2 cluster compared to fumonisin biosynthetic cluster from *F. verticillioides*; B) *M. fijiensis* PKS10-2 cluster compared to solanapyrone biosynthetic cluster from *A. solani*.

A)

| **Gene** | ***M. fijiensis* accession** | ***F. verticillioides* accession** | ***F. verticillioides* gene name** | **Protein sequence similarity** |
| --- | --- | --- | --- | --- |
| PKS | XP_007929903.1 | AAD43562.2 | *FUM1* | 59% |
| Transcription factor | XP_007929904.1 | ABQ95367.1 | *FUM21* | 66% |
| Transporter | XP_007929664.1 | AAN74822.1 | *FUM19* | 51% |
| ɑ-oxoamine synthase | XP_007929637.1 | ADQ39012.1 | *FUM8* | 43% |

B)

| **Gene** | ***M. fijiensis* accession** | ***A. solani* accession** | ***A. solani***  **gene name** | **Protein sequence similarity** |
| --- | --- | --- | --- | --- |
| PKS | XP_007931278.1 | BAJ09789.1 | *sol1* | 52% |
| Dehydrogenase | XP_007928748.1 | BAJ09787.1 | *sol3* | 45% |
| Cytochrome P450 | XP_007931281.1 | BAJ09784.1 | *sol6* | 40% |
| Cytochrome P450 | XP_007931280.1 | BAJ09784.1 | *sol6* | No hits |
