# Supplementary material for: Bioinformatics Prediction of Polyketide Synthase Gene Clusters from Mycosphaerella fijiensis
Source: PLoS One. 2016 Jul 7;11(7):e0158471. doi: 10.1371/journal.pone.0158471 (PMC4936691; doi:10.1371/journal.pone.0158471)
Supplement: S6 Table — Amino acid residues for M. fijiensis PKS sequences and sequences from well-characterized PKS proteins, corresponding to positions 229 and 400 in the fatty acid synthase 1kas. For well-characterized PKS enzymes, the number of iterations catalyzed is shown. Also indicated is whether each PKS is more closely related to the PKS producing 6-methylsalicylic acid (MSAS), naphthopyrone (NAP), or T-toxin (T-tox) (Fig 4), and the number of iterations catalyzed by members of this clade, if known. These are color coded yellow, blue, or pink, respectively. For M. fijiensis PKS sequences in the T-toxin PKS clade, well-characterized PKS enzymes with identical residues at positions 229 and 400 are indicated, along with the number of iterations catalyzed by those PKS enzymes. (DOC) [file pone.0158471.s007.doc]

**S6 Table.** **Amino acid residues for tertiary structure analysis of ketosynthase domains.** Amino acid residues for *M. fijiensis* PKS sequences and sequences from well-characterized PKS proteins, corresponding to positions 229 and 400 in the fatty acid synthase 1kas. For well-characterized PKS enzymes, the number of iterations catalyzed is shown. Also indicated is whether each PKS is more closely related to the PKS producing 6-methylsalicylic acid (MSAS), naphthopyrone (NAP), or T-toxin (T-tox) (Fig 4), and the number of iterations catalyzed by members of this clade, if known. These are color coded yellow, blue, or pink, respectively. For *M. fijiensis* PKS sequences in the T-toxin PKS clade, well-characterized PKS enzymes with identical residues at positions 229 and 400 are indicated, along with the number of iterations catalyzed by those PKS enzymes.

| **PKS** | **229 (1kas)** | **400 (1kas)** | **Closest PKS by homology** | **Actual number of iterations** |
| --- | --- | --- | --- | --- |
| MSAS  *P. griseofulvum* | Y | Y | MSAS (3 iterations) | 3 |
| NAP  *A. nidulans* | Y | A | NAP (5-8 iterations) | 5 |
| Melanin  *B. oryzae* | Y | A | NAP (5-8 iterations) | 5 |
| Melanin  *C. graminicola* | Y | A | NAP (5-8 iterations) | 5 |
| Melanin  *E. dermatitidis* | Y | A | NAP (5-8 iterations) | 5 |
| Melanin  *G. lozoyensis* | Y | A | NAP (5-8 iterations) | 5 |
| Aflatoxin  *A. parasiticus* | Y | A | NAP (5-8 iterations) | 7 |
| Sterigmatocystin  *A. nidulans* | Y | A | NAP (5-8 iterations) | 7 |
| Cercosporin  *C. nicotianae* | Y | A | NAP (5-8 iterations) | 7 |
| Cercosporin  *C. zeae-maydis* | Y | A | NAP (5-8 iterations) | 7 |
| Bikaverin  *F. fujikuroi* | Y | A | NAP (5-8 iterations) | 8 |
| PKS7-1 | Y | A | NAP (5-8 iterations) | Unknown |
| PKS8-1 | Y | A | NAP (5-8 iterations) | Unknown |
| PKS10-1 | Y | A | NAP (5-8 iterations) | Unknown |
| T-toxin  *B. maydis* | Y | Y | T-tox | 20 |
| Compactin  *P. citrinum* | Y | F | T-tox | 8 |
| Lovastatin  *A. terreus* | Y | F | T-tox | 8 |
| Fumonisin  *F. verticillioides* | Y | I | T-tox | 9 |
| Alternapyrone  *A. solani* | F | F | T-tox | 10 |
| Solanapyrone  *A. solani* | Y | Y | T-tox | 8 |
| PKS2-1 | Y | Y | T-tox | Unknown  Same residues as T-toxin and solanapyrone PKS proteins (20 and 8 iterations, respectively) |
| PKS8-2 | Y | I | T-tox | Unknown  Same residues as fumonisin PKS (9 iterations) |
| Hybrid8-3 | Y | F | T-tox | Unknown  Same residues as compactin and lovastatin PKS proteins (both catalyze 8 iterations) |
| PKS8-4 | Y | F | T-tox | Unknown  Same residues as compactin and lovastatin PKS proteins (both catalyze 8 iterations) |
| PKS10-2 | Y | Y | T-tox | Unknown  Same residues as T-toxin and solanapyrone PKS proteins (20 and 8 iterations, respectively) |

ine N-acetyltransferase (Fig 10minedwould be expected to be expressed during infection, though not necessarily when growing sap
