# Supplementary material for: Bioinformatics Prediction of Polyketide Synthase Gene Clusters from Mycosphaerella fijiensis
Source: PLoS One. 2016 Jul 7;11(7):e0158471. doi: 10.1371/journal.pone.0158471 (PMC4936691; doi:10.1371/journal.pone.0158471)
Supplement: S7 Table — Dothideomycete fungal genomes from JGI and NCBI, for tblastn searches for each M. fijiensis PKS sequence, to identify uncharacterized homologs of each PKS. (DOC) [file pone.0158471.s008.doc]

**S7 Table.** **List of Dothideomycete fungal genomes used to identify *M. fijiensis* PKS homologs.** Dothideomycete fungal genomes from JGI and NCBI, for tblastn searches for each *M. fijiensis* PKS sequence, to identify uncharacterized homologs of each PKS.

| **Genomes from JGI** | **Additional Mycosphaerellaceae genomes from NCBI** |
| --- | --- |
| *Aaosphaeria arxii* | *Pseudocercospora pini-densiflorae* |
| *Acidomyces richmondensis* | *Cercospora canescens* |
| *Alternaria alternata* | *Mycosphaerella laricina* |
| *Alternaria brassicicola* | *Mycosphaerella sp. Ston1* |
| *Amniculicola lignicola* | *Mycosphaerella arachidis* |
| *Asplosporella prunicola* | *Lecanosticta acicola* |
| *Aulographum hederae* | *Passalora fulva* |
| *Aureobasidium pullulans var. subglaciale* | *Ramularia collo-cygni* |
| *Aureobasidium pullulans var. melanogenum* | *Mycosphaerella musicola* |
| *Aureobasidium pullulans var. namibiae* | *Mycosphaerella eumusae* |
| *Aureobasidium pullulans var. pullulans* |  |
| *Baudoinia compniacensis* |  |
| *Bimuria novae-zelandiae* |  |
| *Botryosphaeria dothidea* |  |
| *Byssothecium circinans* |  |
| *Cenococcum geophilum* |  |
| *Cercospora zeae-maydis* |  |
| *Cladosporium fulvum* |  |
| *Clathrospora elynae* |  |
| *Cochliobolus carbonum* |  |
| *Cochliobolus heterostrophus C4* |  |
| *Cochliobolus heterostrophus C5* |  |
| *Cochliobolus lunatus* |  |
| *Cochliobolus miyabeanus* |  |
| *Cochliobolus sativus* |  |
| *Cochliobolus victoriae* |  |
| *Corynespora cassiicola* |  |
| *Cucurbitaria berberidis* |  |
| *Decorospora gaudefroyi* |  |
| *Delitschia confertaspora* |  |
| *Didymella exigua* |  |
| *Didymella zeae-maydis* |  |
| *Diplodia seriata* |  |
| *Dissoconium aciculare* |  |
| *Dothidiotthia symphoricarpi* |  |
| *Dothistroma septosporum* |  |
| *Elsinoe ampelina* |  |
| *Eremomyces bilateralis* |  |
| *Glonium stellatum* |  |
| *Hysterium pulicare* |  |
| *Karstenula rhodostoma* |  |
| *Lentithecium fluviatile* |  |
| *Lepidopterella palustris* |  |
| *Leptosphaeria maculans* |  |
| *Lindgomyces ingoldianus* |  |
| *Lizonia empirigonia* |  |
| *Lophiostoma macrostomum* |  |
| *Lophium mytilinum* |  |
| *Macrophomina phaseolina* |  |
| *Macroventuria anomochaeta* |  |
| *Massarina eburnea* |  |
| *Melanomma pulvis-pyrius* |  |
| *Microthyrium microscopicum* |  |
| *Mycosphaerella graminicola* |  |
| *Myriangium duriaei* |  |
| *Mytilinidion resinicola* |  |
| *Neofusicoccum parvum* |  |
| *Ophiobolus disseminans* |  |
| *Paraconiothyrium sporulosum* |  |
| *Patellaria atrata* |  |
| *Phoma tracheiphila* |  |
| *Phyllosticta citriasiana* |  |
| *Piedraia hortae* |  |
| *Pleomassaria siparia* |  |
| *Polychaeton citri* |  |
| *Polyplosphaeria fusca* |  |
| *Pseudovirgaria hyperparasitica* |  |
| *Pyrenochaeta sp. DS3sAY3a* |  |
| *Pyrenophora teres f. teres* |  |
| *Pyrenophora tritici-repentis* |  |
| *Rhizodiscina lignyota* |  |
| *Rhytidhysteron rufulum* |  |
| *Saccharata proteae* |  |
| *Septoria musiva* |  |
| *Septoria populicola* |  |
| *Setomelanomma holmii* |  |
| *Setosphaeria turcica* |  |
| *Sporormia fimetaria* |  |
| *Stagonospora nodorum* |  |
| *Stagonospora sp. SRC1lsM3a* |  |
| *Teratosphaeria nubilosa* |  |
| *Tothia fuscella* |  |
| *Trematosphaeria pertusa* |  |
| *Trichodelitschia bisporula* |  |
| *Trypethelium eluteriae* |  |
| *Venturia inaequalis* |  |
| *Venturia pirina* |  |
| *Verruculina enalia* |  |
| *Westerdykella ornata* |  |
| *Zasmidium cellare* |  |
| *Zopfia rhizophila* |  |
| *Zymoseptoria ardabiliae* |  |
| *Zymoseptoria pseudotritici* |  |
