# Supplementary material for: Bioinformatics Prediction of Polyketide Synthase Gene Clusters from Mycosphaerella fijiensis
Source: PLoS One. 2016 Jul 7;11(7):e0158471. doi: 10.1371/journal.pone.0158471 (PMC4936691; doi:10.1371/journal.pone.0158471)
Supplement: S10 Table — For each RT-PCR assay, primer names, sequences, annealing temperature, extension time, number of cycles, and expected product sizes are indicated. * Same product size expected for cDNA and gDNA products. (DOC) [file pone.0158471.s011.doc]

**S10 Table. Primer sets and conditions for semi-quantitative RT-PCR assays.** For each RT-PCR assay, primer names, sequences, annealing temperature, extension time, number of cycles, and expected product sizes are indicated.

* Same product size expected for cDNA and gDNA products.

| **Gene** | **Forward primer name** | **Forward primer sequence** | **Reverse primer name** | **Reverse primer sequence** | **Annealing temperature** | **Extension time** | **Number of cycles** | **Expected size - cDNA (bp)** | **Expected size - gDNA (bp)** |
| --- | --- | --- | --- | --- | --- | --- | --- | --- | --- |
| Beta-tubulin | q-Tub2l-F | cagctcgagcgcatgaacg | qTub1l-R | ggtgcgaaaccgaccatgaag | 59 | 1:00 | 40 | 745* | 745* |
| PKS2-1 | PKS2-1 1F | atggcagtcaataccgatga | PKS2-1 450R | gcttcgtatgagacttccag | 53 | 0:45 | 45 | 344 | 450 |
| PKS7-1 | PKS7 659F | gccgtctatgactattgaca | PKS7 1413 R | tggctgttgattacgctctt | 52 | 1:30 | 45 | 660 | 774 |
| PKS8-1 | PKS8-1 1685F | caggacgcatcaactacttc | PKS8-1 2176R | ctcggcggagtggttagttc | 54 | 0:45 | 45 | 362 | 511 |
| PKS8-2 | PKS8-2 5626F | caggaagattgacgaaaggc | PKS8-2 6160R | catagtgttggatcatgtcg | 52 | 0:45 | 45 | 524 | 574 |
| Hybrid8-3 | PKS8-3 8536F | ctcgccgaacttgatggaga | PKS8-3 9101R | tacaggcatcggaacgacgagg | 59 | 1:30 | 45 | 495 | 585 |
| PKS8-4 | PKS8-4 656F | tatgctctcacctgacgg | PKS8-4 1524R | agattcatagctctcgat | 47 | 1:30 | 45 | 542 | 886 |
| PKS10-1 | PKS10-1 2570F | ttctcatacacgaccactgc | PKS10-1 3153R | gcaagcaatctcggtcatcttg | 55 | 0:45 | 45 | 603* | 603* |
| PKS10-2 | PKS10-2 367F | cttcgtgtttcatagggaac | PKS10-2 1156R | catcaacttcaatcggatcg | 51 | 1:30 | 45 | 615 | 809 |
